# The effects of an integrated exercise and cardiovascular health education programme on community-dwelling older adults at risk of atherosclerotic cardiovascular diseases: A study protocol for a randomised controlled trial

**DOI:** 10.1371/journal.pone.0286181

**Published:** 2023-05-24

**Authors:** Flora M. W. Lo, Eliza M. L. Wong, Ka Yan Ho

**Affiliations:** 1 Faculty of Health and Social Sciences, Hong Kong Polytechnic University, Hung Hom, Hong Kong SAR, China; 2 School of Nursing, Tung Wah College, Mongkok, Hong Kong SAR, China; 3 School of Nursing, Hong Kong Polytechnic University, Hung Hom, Hong Kong SAR, China; Kurume University School of Medicine, JAPAN

## Abstract

**Background:**

Although older adults are at an increased risk of atherosclerotic cardiovascular disease (ASCVD), the effect of an integrated exercise and cardiovascular health education programme based on self-efficacy theory has not been well investigated among older adults. This study aims at examining the effect of this programme on community-dwelling older adults at risk of ASCVD concerning physical activity level, exercise self-efficacy and ASCVD risk profile.

**Methods:**

A parallel two-arm randomised controlled trial with pretest-posttest design will be performed among 190 Chinese community-dwelling adults aged 60 or above in elderly community centres of the Guangdong-Hong Kong-Macao Greater Bay Area. Eligible participants will be randomised by computerised generation. Experimental group will receive a 12-week integrated exercise and cardiovascular health education programme, which comprises a one-hour group-based health education talk conducted at Week 1, a booklet, a lecture video, a tailor-made exercise video, and a booster intervention by text messaging starting from Week 1 to Week 12. Control group will receive placebo intervention including a talk on basic health issues, a lecture video and corresponding leaflet. The outcomes will be investigated through self-report questionnaires and physiological evaluations at baseline, Week 12, Week 24, and Week 36. Physical activity level, exercise self-efficacy and ASCVD risk profile will be assessed, with physical activity level at Week 24 considered the primary outcome. The main intervention effect (group differences on continuous outcome variables) will be examined via Generalized Estimating Equations with identity link.

**Discussion:**

This study findings will provide clues to the effect of the integrated exercise and cardiovascular health education programme, which is theoretically underpinned with self-efficacy theory, in older adults at risk of ASCVD. It will also enhance the quality of community health education by providing insight into the effective teaching strategies targeting older adults.

**Trial registration:**

This study has been registered on ChinicalTrial.gov (Trial ID: NCT05434273).

## Introduction

Atherosclerotic cardiovascular diseases (ASCVDs), which are cardiovascular diseases caused by plague formation in the arteries, represent approximately 85% of global cardiovascular mortality in 2016 [1]. ASCVDs mainly include coronary heart disease and stroke as they share common aetiologies and risk-enhancing factors including type II diabetes, hypertension, dyslipidaemia, unhealthy diet, physical inactivity, smoking, obesity, and family history [2, 3].

Older adults are exposed to an increased ASCVD risk. Approximately 80% of coronary heart disease mortality in America are older adults while 17% of stroke patients are aged over 85 years [4, 5]. In China, the 10-year ASCVD predicted risk for older adults was projected to be ranged from 5% to 20%, and higher risk was indicated in those with hypertension, diabetes, or smoking habit [6]. Physical activity (PA) is effective in preventing ASCVDs via constraining its risk-enhancing factors including hypertension, diabetes, and obesity [7]. Nonetheless, older adults are the least physically active [7, 8], with less than 60% of them meet the PA recommendation suggested by the World Health Organisation [9]. Hence, it is crucial for healthcare professionals to promote the PA levels of older adults with an ultimate goal to prevent them from developing ASCVDs.

A review of literature identified several studies which examined the effect of various health education programmes in promoting PA among older adults who were at risk of ASCVDs. In these programmes, health information was mostly delivered in a lecture format, accompanied with written materials, i.e. booklet. PA components were also included in previous studies, in which two provided PA advice [10, 11] and six incorporated exercise class in their programmes [12–17]. Supporting strategies such as cardiovascular risk evaluations, motivational strategies or goal setting were also employed [10–14, 16, 17]. These studies had demonstrated positive effects in reducing within-group blood pressure (BP). However, their overall results were not promising as the programme effects on PA level, between-group BP, Body Mass Index (BMI), weight, and waist circumference remained inconclusive [10–13, 15–17]. Moreover, suboptimal attendance rates were common in most studies with exercise classes [12, 13, 15–17]. Resnick et al. [17] revealed that participants were relatively non-adherent to the programme, with merely 33% of participants joined 90% of sessions. For exercise class, the attendance rate for each session was only 60%. Likewise, Amundson et al. [12] and Brokaw et al. [13] demonstrated relatively low attendance rates (14.5 and 13.7 sessions out of 22 sessions respectively). The suboptimal exercise adherence was echoed in a systematic review by Picorelli et al. [18], which indicated that the attendance rates of exercise sessions among older adults ranged from 58% to 77% in five included studies.

A possible reason for the suboptimal exercise adherence is the lack of motivation among older adults. A systematic review by Room et al. [19] revealed that exercise adherence of older adults could be adversely affected by low self-efficacy. Such inference was consistent with another review by Rivera-Torres et al. [20], which stated that self-efficacy played a crucial role in exercise adherence among older adults and suggested to embed the concept of self-efficacy in the design of intervention programmes. However, most existing health education programmes did not aim to enhance the exercise self-efficacy of older adults and did not incorporate self-efficacy theory in the programme development [12, 13, 15, 16]. In fact, increasing number of studies have shown that self-efficacy is a core motivator of PA among older adults [21, 22].

According to Bandura and Adams [23], self-efficacy is defined as a person’s perception in his/her own capability of attaining something successfully [24]. It is postulated that a person with stronger perceived self-efficacy is more likely to perform behaviours needed in attaining specific objectives, and inclines to be more persistent and proactive in overcoming challenges [23]. This concept has been widely applied in studies on cardiac rehabilitation and self-management. The results from these studies have indicated that health education programme based on self-efficacy concept could greatly improve lifestyle and self-care behaviours in older adults [25–27].

Apart from the aforementioned issue, there are several limitations in the existing studies on the health education programme in PA for older adults at risk for ASCVDs. Firstly, these studies were exploratory in nature, with most using either a quasi-experimental or a one group study design [10–14,16, 17]. Secondly, most of them were small-scale, with their sample size ranging from 11 to 46 [10, 14, 17]. Thirdly and perhaps most importantly, some studies only targeted on few risk factors of ASCVDs, for instance, nutrition or PA, and overlooked the fact that the aetiology of ASCVDs is multifactorial [14, 16]. Given the above issues, an integrated exercise and cardiovascular health education programme targeting multiple risk factors, together with increased PA levels based on self-efficacy concept seems to offer a better outcome for older adults at risk of ASCVDs [28]. However, to the best of our knowledge, there are limited studies which examine the effect of such an integrated programme among older adults in terms of their PA levels, self-efficacy, and ASCVD-related outcomes. Hence, more empirical studies are required.

### Theoretical framework

The integrated exercise and cardiovascular health education programme is guided by the self-efficacy theory advocated by Bandura and Adams [23]. In this theory, self-efficacy is a core which affects choice of activities, effort, persistence, and achievement. It is postulated that people with high self-efficacy opt to participate in activities more readily, with greater efforts and longer duration. Four factors are shown to be able to increase self-efficacy: mastering experience, vicarious experience, verbal persuasion, and emotional states [24]. Mastering experience refers to the experience a person attains when taking on a new activity and successfully managing it; while vicarious experience means that observing people similar to oneself succeed strengthens the observer’s beliefs that he or she gets the same capability to master similar activities. In addition, social persuasion emphasizes on positive feedbacks received from others to enhance one’s own belief of success. Emotional states mean that a person experiences sensational change regarding his or her body, and the way this emotional arousal is perceived can influence his or her self-efficacy. Based on this theory, we will incorporate these four components in our integrated exercise and cardiovascular health education programme, thus enhancing older adults’ self-efficacy in engaging in PA (details are shown in the “Materials and methods” section). It is hypothesised that older adults will eventually adopt, maintain, and adhere to PA and other healthier lifestyle behaviours, thereby lowering ASCVD risks.

## Materials and methods

### Study aims

This randomised controlled trial aims to examine the effect of the integrated exercise and cardiovascular health education programme for community-dwelling older adults at risk of ASCVD on PA level, exercise self-efficacy, and ASCVD-related outcomes.

### Study design and setting

**Fig 1:**
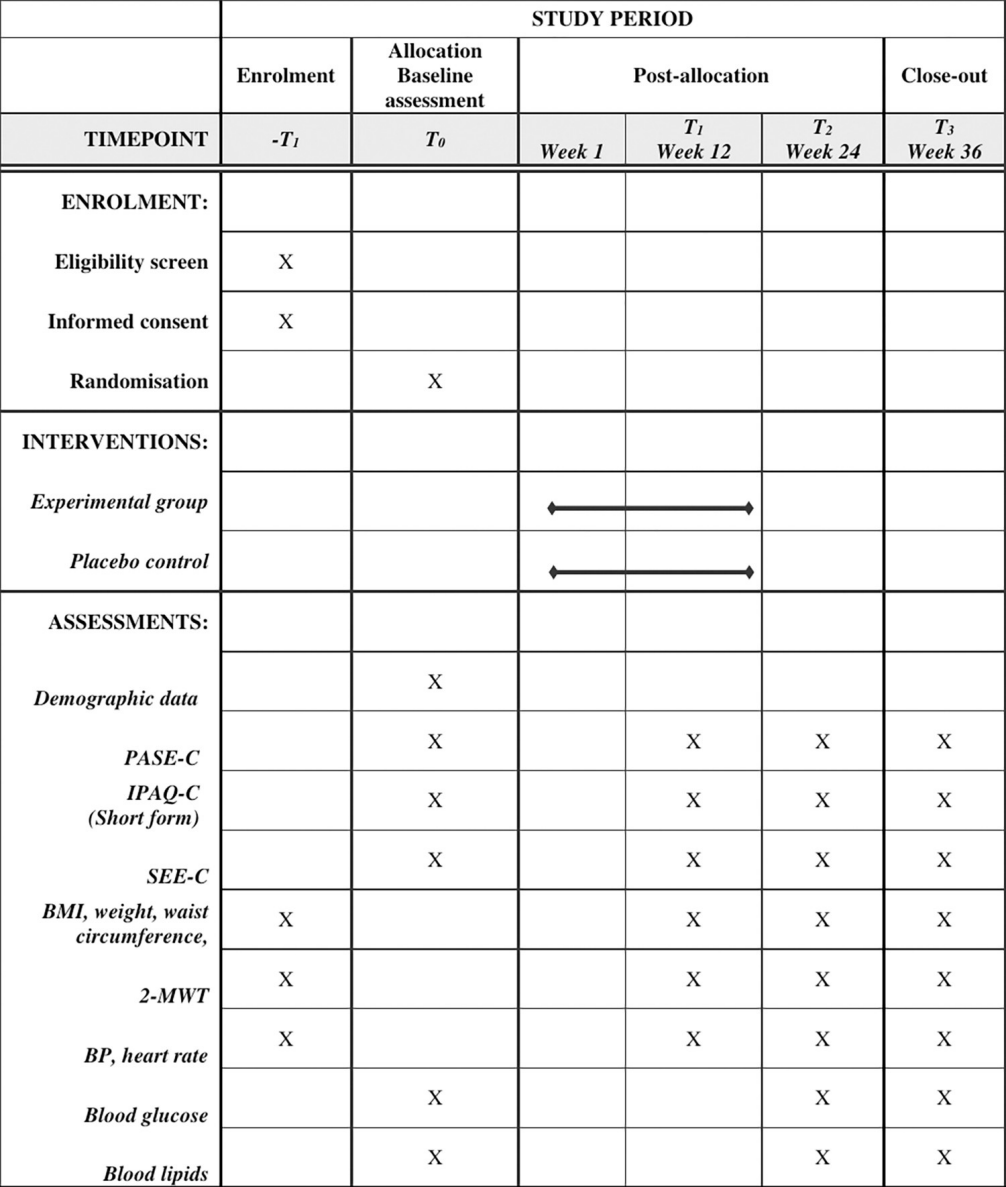
Schedule of enrolment, interventions, and assessments. Abbreviations: Two-Minute Walk Test (2-MWT), Body Mass Index (BMI), blood pressure (BP), Chinese version of International Physical Activity Questionnaire (short form) (IPAQ-C, short form), Chinese version of Physical Activity Scale for the Elderly (PASE-C), Chinese version of Self-Efficacy for Exercise (SEE-C).

The protocol will be reported following the SPIRIT reporting guideline [29]. We will adopt a randomised controlled trial design. This trial will be conducted in urban elderly community centres in the Guangdong-Hong Kong-Macao Greater Bay Area. Fig 1 shows the schedule of enrolment, interventions, and assessments.

### Participants

Community dwelling adults will be eligible if they fulfil these inclusion criteria: (1) aged 60 years old or above [30–32]; (2) having at least one ASCVD risk factor, including current smoking, excessive alcohol consumption (daily intake of more than one drink for women and more than two drinks for men), being diagnosed with hypertension, diabetes mellitus or hyperlipidaemia, overweight (BMI which is equal or greater than 25 kg/m^2^), family history of coronary heart disease/stroke or anticoagulant use for ASCVD prevention [1, 33–36]; (3) passing the cardiovascular fitness test; (4) having a mobile and able to read text messages, and (5) able to read Chinese and communicate in Cantonese. However, we will exclude older adults who (1) are visually impaired, hearing impaired, or suffering from any cognitive, psychiatric, or musculoskeletal disorder; (2) have a history of attending any cardiovascular education programme; or (3) have a medical diagnosis of coronary heart disease or stroke.

### Sample size

Taking PA level as the primary outcome, with reference to a meta-analysis by Conn et al. [37], the overall mean weighted effect size of patient education on PA level among 17147 patients with chronically diseases is 0.45. The sample size of the proposed study is calculated via G*Power calculator [38]. For a two-tailed test, with the desired statistical power of 0.80 and significance level of 0.05, the estimated sample size is 158. Although the attrition rates of five related studies were less than 20% [11, 12, 14, 15, 17], for conservative reason, an attrition rate of 20% is assumed. Hence, a total of 190 participants (95 participants per group) will be recruited in the study.

### Randomisation, blinding, and concealment

**Fig 2:**
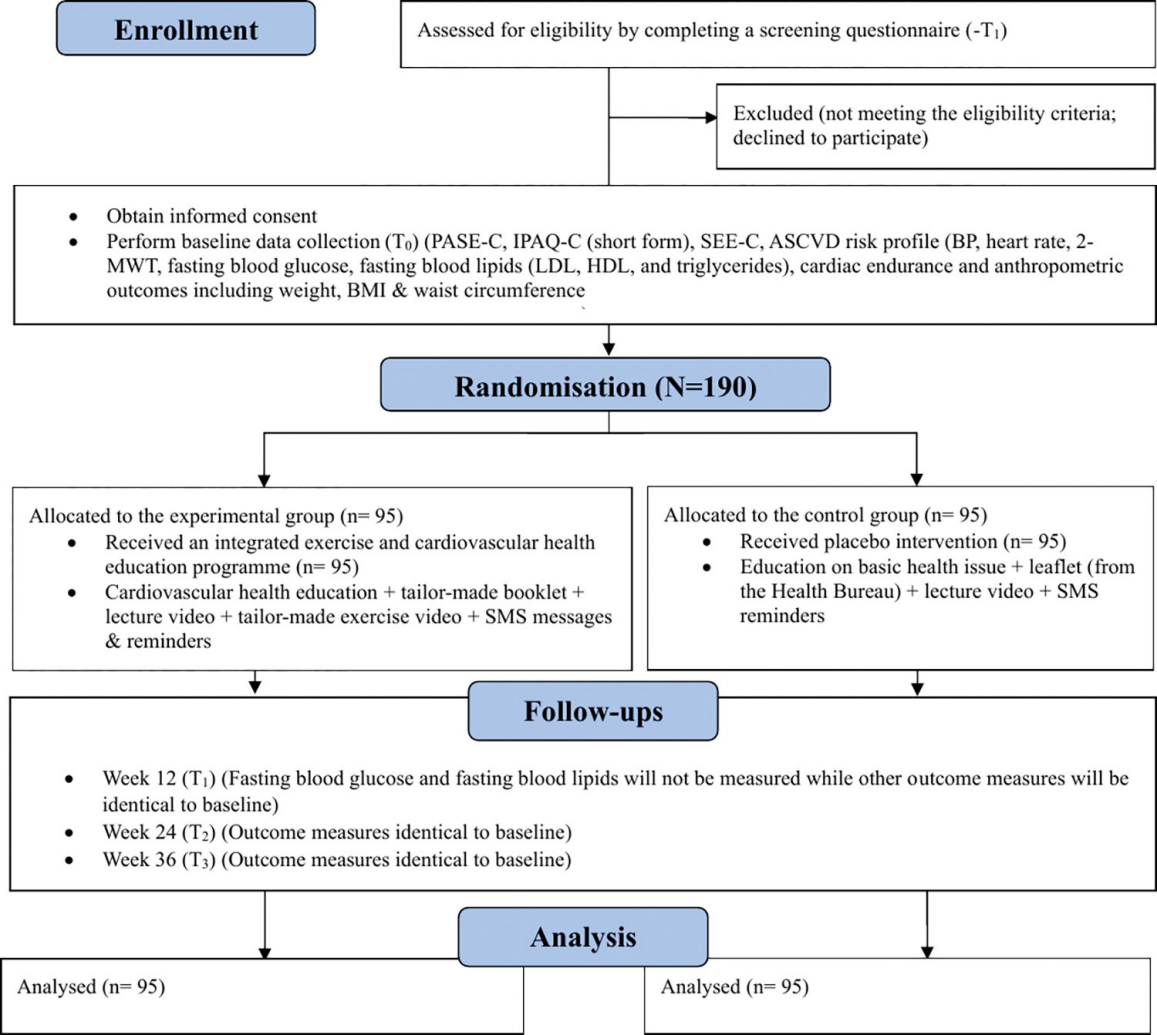
Flow diagram of the study. Abbreviations: Two-Minute Walk Test (2-MWT), atherosclerotic cardiovascular disease (ASCVD), Body Mass Index (BMI), blood pressure (BP), Chinese version of International Physical Activity Questionnaire (short form) (IPAQ-C, short form), Chinese version of Physical Activity Scale for the Elderly (PASE-C), Chinese version of Self-Efficacy for Exercise (SEE-C), high-density lipoprotein (HDL), low-density lipoprotein (LDL), short message service (SMS).

Following the completion of informed consent and baseline data collection, eligible participants will be allocated into either experimental or control group with 1:1 allocation ratio by a computerised random number generator (Fig 2). The random allocation sequences will be generated by a research assistant not engaging in recruitment. The sequences will be put in sequentially numbered, opaque, sealed envelopes [39, 40]. Research assistants will draw the envelopes in sequence to determine participants’ group allocation. To minimise performance bias, research assistants will not acknowledge participants their group assignment. To avoid contamination, the two groups will receive their corresponding treatments at different times in a private room. In addition, the two groups will undergo data collections at different times. Participants will be instructed not to share their education materials to others following the intervention. To avoid detection bias, the research assistant who performs data collection will be blinded to the group assignment. Since the principal investigator (a nurse) is responsible for conducting the lecture of the experimental group, she is unlikely to be blinded.

### Intervention

#### (1) Experimental group

A systematic review by Peiris et al. includes 11 studies which examined the effect of lifestyle intervention programme integrated with education and unsupervised PA among adults with metabolic syndrome [41]. The intervention period of these studies ranged from 12 to 144 weeks and the duration of at least three months enhanced the outcomes significantly. Hence, the intervention period will last 12 weeks. Participants in the experimental group will receive a self-efficacy-based, integrated exercise and cardiovascular health education programme conducted by the primary research nurse for 12 weeks. The intervention comprising a health education talk, a booklet, a tailor-made exercise video, a lecture video, and a set of one-way text messages is designed based on the self-efficacy theory and is validated by an expert panel which contains two associate professors, one assistant professor, one physiotherapist, one cardiac nurse specialist, one cardiologist and one registered dietitian, with all of whom having considerable experience in conducting studies on lifestyle modification. Contents of the talk are derived from regional and international guidelines on PA recommendation and ASCVD prevention [34, 35, 42–46]. The components of ASCVD prevention are based on the seven predictors of cardiovascular health included in the Life’s Simple 7, a prescription for health formulated by the American Heart Association [42]. While PA will be emphasised in the talk, other elements of ASCVD prevention such as healthy diet, smoking cessation, as well as management on weight, BP, cholesterol, and blood glucose will also be introduced. In Week 1, a nurse will deliver a 60-minute face-to-face health education talk to a group of four to six participants. During the talk, the nurse will educate the participants about the risk factors of ASCVD. Apart from that, each participant will be invited to share his or her experience of performing PA and offer comments to other participants (vicarious experience). During the discussion, the nurse will offer positive feedbacks based on their sharing (verbal persuasion). If the participants raise some negative experience or feelings in relation to PA, suggestions on how they can do PA better will be provided to change their perceptions and feelings (emotional states). In the end, the nurse will demonstrate a set of exercises tailor-made for the elderly and ask the participants to do the return demonstration (mastery experience). These simple exercises are at moderate-intensity and involve an integration of balance training, aerobic and muscle-strengthening activities using a chair and a towel. To fulfil the PA recommendations suggested by the World Health Organisation (a minimum of either 150-minute aerobic PA with moderate intensity or 75-minute aerobic PA with vigorous intensity or a proportional combination of both every week) [7], participants will be encouraged to perform these exercises twice a day, 25 minutes each time in which 5-minute warm-up and 5-minute cool down exercises will be included. To enhance the standardisation of intervention delivery, a tailor-made exercise video, in which a nurse and two elders taking the lead in performing the aforementioned exercises, will be displayed during exercise demonstration (mastery experience and vicarious experience). The exercise video begins with a series of mobility and stretching exercises as warm-up, followed by a series of fitness exercises, including chair aerobics (e.g. hip marching, arm raises, arm swings, and chest expansions), balance exercises (e.g. single-leg stance, and single-leg swings), muscle-strengthening exercises (e.g. sit to stand, calf raises, and leg extension) and towel exercises. The video ends with cool-down exercises which comprise breathing and stretching exercises. To facilitate participants to revise the education materials and practice the exercises at home, each of them will receive a 17-inch portable video player which contains a pre-recorded lecture video (containing the same content as the health education talk) and an exercise video following the talk (mastery experience). On the other hand, a booklet which contains the key points of the health education talk will be provided for the participants as reference (mastery experience). A telephone number will also be provided for the participants in case they have any question or worry regarding the programme (emotional states). To improve exercise adherence, experimental group will be required to document their PA patterns in an exercise log daily.

After the health education talk, a booster intervention will be provided for the participants via short message service (SMS) for 12 weeks to sustain older adults’ motivation in engaging in PA across the study period. Text messaging was adopted in previous behavioural interventions targeting older adults and was shown to have positive outcomes in terms of increased PA and better medical compliance [47, 48]. The SMS we used is one-way as older adults generally consider this communication method to be encouraging and able to remind them important messages [47–49]. As comparable benefits were demonstrated between high-frequency (more than one message per day) and low-frequency messaging (three to five messages per week) [50], it will be appropriate to adopt three messages per week to maintain participants’ motivation while minimising the annoyance of receiving excessive messages. Hence, a total of 36 SMS messages (one message on every Monday, Wednesday, and Friday) will be delivered in the daytime (from 9am to 5pm), according to the participants’ preference. The content of the messages is modified from the study by Müller et al. [47], and will include greeting, instruction/encouragement section and closing. Meanwhile, the message theme is derived from the components of self-efficacy theory including mastering experience, verbal persuasion, and emotional states, with each being used rotationally in each message. The message covers key points of the education talk, encouraging words, reminders, and potential barriers of performing PA and corresponding coping strategies. At Week 7, the participants will receive a SMS message which contains a progress report regarding their interim performance on PA with corresponding advice and feedback (verbal persuasion and emotional states).

#### (2) Control group

A placebo-controlled intervention will be provided to participants in the control group. Particularly, they will receive a 60-minute face-to-face health education talk on basic health issues. Information regarding PA and cardiovascular health education will not be provided during the talk. A 17-inch portable video player which contains a pre-recorded lecture video regarding the placebo health education talk and a corresponding governmental leaflet will be delivered as reference. Aside from receiving SMS reminder messages on attending the talk and data collection, no other SMS messages will be sent.

The primary research nurse will be responsible for preparing the education materials for both groups. Also, the health education talk of the experimental group will be conducted by the primary research nurse. For the control group, the talk will be conducted by another registered nurse. To ensure standardised lecture delivery, a training session will be provided to the nurse who engages in lecture delivery of the control group. Also, 10% of the intervention will be audio-recorded for quality control. To reduce subject contamination, the talk will be conducted in a private room and the two groups will receive their session at separate times.

### Data collection

For the consideration of data collection period, the systematic review by Peiris et al. indicated that the timing of outcome measurement among 11 included studies regarding lifestyle modification ranged from 12 to 144 weeks, with 24 weeks being mostly adopted in the effective studies [41]. Hence, in this study, the outcomes will be measured at baseline and Week 24, with extra measures at Week 12 and Week 36 added to serve as the interim and follow-up evaluations respectively. Convenience sampling will be employed for subject recruitment. A recruitment poster will be displayed in the elderly community centres. Older adults who are interested to join the study can contact the staff at the centres who will subsequently make referral to the research team. In case of having activities in which many older adults will be involved, the principal investigator will be present at the elderly centres to promote the programme. Potential participants will be approached by a research assistant face-to-face and participant eligibility will be screened via completing a structured eligibility checklist in the elderly community centres (-T_1_). After confirming the eligibility and providing the study’s details, written consent will be obtained and baseline data (T_0_) will be collected. After that, participants will be randomly assigned to either experimental or control group and will subsequently receive corresponding treatments. Participants will complete three follow-ups face-to-face at the elderly community centres at Week 12 (T_1_), Week 24 (T_2_), and Week 36 (T_3_). To ensure data reliability, research assistants who engage in data collection will receive a training session on the entire data collection procedure, procedures of physiological assessments and ways of assisting participants in completing questionnaires. Training PowerPoint slides will be provided to outcome assessors after the training session for reference.

### Outcomes

The effects of the integrated health education programme on PA level, exercise self-efficacy, and ASCVD-related outcomes will be examined. PA level throughout 24 weeks, which is measured with the Chinese version of the Physical Activity Scale for the Elderly, will be the primary outcome while others will be secondary outcomes.

#### (1) The Chinese version of the Physical Activity Scale for the Elderly (PASE-C)

The participants’ total PA level will be quantified with the PASE-C because of its comprehensiveness in measuring PA in various settings and its specificity for the elderly. This questionnaire will ask the participants to report the leisure, physical, household, and work-associated activities in the past seven days [51]. The PASE-C has been used in Asian populations aged 60 or above [52, 53] and possesses satisfactory test-retest reliability [54].

#### (2) The Chinese version of the International Physical Activity Questionnaire (short form) (IPAQ-C)

This questionnaire will document self-reported physical activity in the last seven days. Based on the collected data, participants can be classified into “inactive”, “minimally active” or “health enhancing physical activity active” via calculating the number of days and the total volume of PA by intensity. This questionnaire is widely used, cost-effective, possesses satisfactory reliability and comparable psychometric properties with the long form [55–57].

#### (3) The Chinese version of Self-Efficacy for Exercise (SEE-C)

This scale will be used to assess the participants’ self-efficacy to perform PA. The psychometrics of the SEE-C has been assessed in Chinese populations, with satisfactory internal consistency [58, 59].

#### (4) ASCVD-related outcomes

We will assess the following ASCVD-related outcomes: BP, fasting blood glucose, fasting blood lipids (low-density lipoprotein cholesterol, high-density lipoprotein cholesterol, and triglycerides), weight, BMI, waist circumference, and cardiac endurance. BP will be measured in a sitting position using calibrated, automatic BP monitor for two times. Participants will take a 5-minute rest prior to the measurement and 1-minute interval will be provided between each measurement [60]. Average of two measurements will be reported [11, 16]. Height will be recorded using a stadiometer while weight will be assessed using a calibrated scale, with results corrected to the nearest 1 cm and 0.1 kg respectively. Waist circumference will be measured two times in a standing position at the midpoint between the upper point of the iliac crest and the bottom of the last rib using a soft measuring tape [61]. Average of two measurements will be reported and corrected to the nearest 0.1 cm. Blood glucose and blood lipids will be measured via obtaining finger-prick blood samples using an auto-analyser. Participants are required to fast 8 hours before obtaining blood samples. A Two-Minute Walk Test will be used to assess the participants’ cardiac endurance. We will record the maximum distance that the participants can cover in two minutes [62]. This test is easy to implement and possesses satisfactory and comparable psychometric properties with a Six-Minute Walk Test, Timed Up and Go Test and Berg Balance Scale among older adults [63–65].

The evaluation results of BP, heart rate, Two-Minute Walk Test, BMI, weight, and waist circumference assessed at the screening stage will be included as the baseline data. As the systematic review by Peiris et al. [41] revealed that it took more than 3 months for lifestyle modification programmes integrated with unsupervised PA to cause significant impacts on fasting blood glucose, while another systematic review by Aucott et al. [66] indicated that lifestyle modification programmes targeted at weight loss might have positive impacts on long-term lipid profile, these two outcomes will be both measured at baseline (T_0_), Week 24 (T_2_), and Week 36 (T_3_) only.

### Data analysis

The data collected will be primarily analysed by SPSS version 26. The baseline demographic and clinical characteristics of the participants in the experimental and control groups will be presented in a table. For continuous variables with normal distribution, the data will be summarised by mean and standard deviation whereas median and interquartile range will be used in the report of continuous data with non-normal distribution. Meanwhile, categorical variables will be summarised for each group by proportion and frequency. With reference to existing studies, age was suggested to be a prognostic factor of PA level, lipid level, weight, and BMI [12–14] while gender might have prognostic importance on PA and lipid levels [12, 14]. Also, hypertension was suggested to be influential to weight [12]. These baseline variables will be regarded as covariates and will be adjusted in the analysis. Meanwhile, as multiple community elderly centres will be involved in the study, centre will also be regarded as a covariate owing to the variations in contextual factors [10].

Generalised estimating equations (GEE), which is an extension of the generalised linear models, is prevalently used in studies with repeated measurements. This semi-parametric method is comparatively flexible in a way that it works with different kinds of data (such as continuous, binary and count data) by applying different link functions [67]. Also, GEE is relatively robust regardless of the choice of working correlation matrix [67]. In this study, for comparing group differences on continuous outcome variables (PA score quantified with PASE-C, exercise self-efficacy score, BP, weight, BMI, waist circumference, fasting glucose, fasting blood lipids, and distance walked), GEE with identity link will be used. For ordinal outcome variables (PA level quantified with IPAQ-C), GEE with cumulative logit link will be used. P-value of less than 0.05 will be regarded as statistically significant. The selection of working correlation matrix will depend on the correlation structure of the observed data. The Quasi-likelihood under Independence Model Criterion (QIC) for the model with each working correlation matrix will be compared and the matrix with the smallest QIC will be preferred [68].

In case a continuous outcome variable appears to be skewed and is defined on a positive support, suitable Generalized Additive Models for Location, Scale and Shape (GAMLSS) will be built using the gamlss package in R (version 4.1.0.) to explore the programme effectiveness on this variable. GAMLSS are convenient, flexible statistical models that can be used to deal with data with numerous types of distributions, including those with highly skewed or/and kurtotic distributions [69]. In this case, a suitable distribution for the outcome variable will be selected by comparing different positively skewed continuous distributions in R+, while taking kurtosis into consideration. A suitable GAMLSS model will be chosen through making comparisons between numerous competing models derived from various combinations of their components [69]. The diagnostics for GAMLSS models will be carried out by means of diagnostic plots (including worm-plots and residual plots).

As the study will last 36 weeks and will involve four data collection timepoints, it is possible that the absence rate may increase in subsequent post-tests. Also, dropout owing to scheduling conflict, change in health status or loss to follow-up may be another concern. These situations result in the occurrence of missing data which may further threaten the validity of the study. The most essential way of minimising the effect of missing data is to ensure participants remain in the study until all outcome data are collected [70]. In the study, a reminder message will be sent to remind participants the evaluation arrangement. Additionally, the primary research nurse will maintain close contact with the centre staff to ensure that the participants can be contacted if they move out. Reasons for dropout will also be recorded. Meanwhile, intention-to-treat analysis will be used to reduce bias related to the non-random loss of participants. Missing data owing to dropouts or incomplete measurements will be treated as missing and analysed via missing value analysis. Little’s Missing Completely at Random (MCAR) test will be conducted to provide clues as to whether the data are MCAR. Meanwhile, the relationship between the missingness on one variable and the values of other variables will be examined to see if the data are potentially missing at random (MAR). Also, the reasons for dropout will be investigated. The aforementioned GEE analysis will be adopted under MCAR assumption whereas weighted GEE analysis will be employed when data are MAR [70]. For the data which are missing not at random (MNAR), controlled imputation methods will be used [70].

### Ethics and dissemination

The study has been registered on the ChinicalTrial.gov (NCT05434273) and has obtained the ethical approval from the Human Subjects Ethics Sub-committee of the Hong Kong Polytechnic University (HSEARS20220728001). Eligible participants will be acknowledged about the research safety and their rights of participations, withdrawals, and refusals in providing information. They will be given research information sheets and sufficient time for consideration before signing written consents. All participant-related information, questionnaires and consent forms will be kept in locked cabinets with limited access. Participants’ identity will be concealed via coded identification numbers. Meanwhile, databases will be encrypted and only research team has granted the access right.

## Discussion

This study will provide clues regarding the effect of an integrated exercise and cardiovascular health education programme guided by self-efficacy theory on exercise self-efficacy, PA levels and ASCVD-related outcomes among community-dwelling older adults at risk of ASCVD. In fact, the healthcare burden associated with ASCVDs is enormous and long-term. Health education is always regarded as one of the most cost-effective ways for prevention of ASCVDs among older adults in the community. If our self-efficacy-based intervention is proven to be effective in promoting PA for older adults, this can inform healthcare professionals the more effective teaching strategy when compared to the traditional approach. This teaching strategy can subsequently be applied to other health education programmes for older adults, thus motivating them to adopt a healthy lifestyle, with an ultimate goal of preventing ASCVDs among this cohort in the long-run.

Despite the programme being evidence-based and theory-driven, three limitations may likely arise. First, as the programme is conducted in Cantonese and Chinese, study applicability may be limited as foreign participants are excluded. Second, as participants need to undergo multiple data collection procedures, a higher absence rate may exist in the post-tests. Hence, a reminder message will be sent to remind participants the evaluation arrangement. Third, although several precautions have been made to prevent contamination, there is a potential risk that participants may know other group’s allocated treatment as they may be from the same centre.

## Supporting information

S1 File SPIRIT checklist. (DOCX)

S2 File Study protocol. (DOCX)
