# Supplementary material for: The effects of an integrated exercise and cardiovascular health education programme on community-dwelling older adults at risk of atherosclerotic cardiovascular diseases: A study protocol for a randomised controlled trial
Source: PLoS One. 2023 May 24;18(5):e0286181. doi: 10.1371/journal.pone.0286181 (PMC10208471; doi:10.1371/journal.pone.0286181)
Supplement: S2 File — (DOCX) [file pone.0286181.s002.docx]

**The Effects of an Integrated Exercise and Cardiovascular Health Education Programme on Community-Dwelling Older Adults at Risk of Atherosclerotic Cardiovascular Diseases: A Randomised Controlled Trial**

**Abstract**

**Background:** Although older adults are at an increased risk of atherosclerotic cardiovascular disease (ASCVD), the effect of an integrated exercise and cardiovascular health education programme based on self-efficacy theory has not been well investigated among older adults. This study aims at examining the effect of this programme on community-dwelling older adults at risk of ASCVD concerning physical activity level, exercise self-efficacy and ASCVD risk profile.

**Methods:** A parallel two-arm randomised controlled trial with pretest-posttest design will be performed among 190 Chinese community-dwelling adults aged 60 or above in elderly community centres. Eligible participants will be randomised by computerised generation. Experimental group will receive a 12-week integrated exercise and cardiovascular health education programme, which comprises a one-hour group-based health education talk conducted at Week 1, a booklet, a lecture video, a tailor-made exercise video, and a booster intervention by text messaging starting from Week 1 to Week 12. Control group will receive placebo intervention including a talk on basic health issues, a lecture video and corresponding leaflet. The outcomes will be investigated through self-report questionnaires and physiological evaluations at baseline, Week 12, Week 24, and Week 36. Physical activity level, exercise self-efficacy and ASCVD risk profile will be assessed, with physical activity level at Week 24 considered the primary outcome.

**Significance:** This study findings will provide clues to the effect of the integrated exercise and cardiovascular health education programme, which is theoretically underpinned with self-efficacy theory, in older adults at risk of ASCVD. It will also enhance the quality of community health education by providing insight into the effective teaching strategies targeting older adults.

**Introduction**

**Background**

Atherosclerotic cardiovascular diseases (ASCVDs), which are cardiovascular diseases (CVD) caused by plague formation in the arteries, represent 85% of global cardiovascular (CV) mortality in 2016 (World Health Organization [WHO], 2017). ASCVDs generally include coronary heart disease (CHD) and stroke as they share common aetiologies and risk-enhancing factors including type II diabetes, hypertension, dyslipidaemia, unhealthy diet, physical inactivity, smoking, obesity, and family history (ARUP Consult, 2020; Zhao et al., 2019).

Older adults are exposed to an increased ASCVD risk. Approximately 80% of CHD deaths in America were older adults while 17% of stroke patients aged over 85 (Benjamin et al., 2019; Mozaffarian et al., 2016). In China, the 10-year ASCVD predicted risk for older adults ranged from 5% to 20%, and higher risk was indicated in those with hypertension, diabetes, or smoking habit (WHO, 2019). However, older adults are often neglected in ASCVD prevention owing to the fallacy that they are not benefited much from preventive measures (Barry et al., 2016). They are underrepresented in CV guidelines, trials and ASCVD prevention programmes (Konrat et al., 2012; Sardar et al., 2014). Hence, promoting ASCVD prevention among older adults is imperative.

Physical activity (PA), which refers to motion or movement generated by skeletal muscles though exergy utilisation, is effective in preventing ASCVD via constraining hypertension, diabetes, and obesity (WHO, 2020). WHO (2020) recommends that older adults should engage in a minimum of either 150-minute aerobic PA with moderate intensity or 75-minute aerobic PA with vigorous intensity or a proportional combination of both every week. Additionally, multicomponent PA of at least moderate intensity (strength training and functional balance), is suggested to be performed at least three times per week. Nonetheless, older adults are the least physically active compared with other age groups (Bull et al., 2004; WHO, 2020), with less than 60% met WHO’s PA recommendation in most studies (Sun et al., 2013). Hence, increasing PA among older adults is imperative.

Notably, self-efficacy is a core motivator of increased PA among older adults (Cavill and Foster, 2018; McAuley et al., 2011). First, self-efficacy is essential in PA initiation. Second, self-efficacy has positive influence on PA maintenance of older adults (McAuley et al., 2003, 2007; White et al., 2012), which helps to achieve sustainable impact on ASCVD prevention (Marcus et al., 2000; Warburton et al., 2006). Conversely, insufficient provision of PA information and professional guidance are barriers of older adults in engaging PA (Bethancourt et al., 2014; Cavill and Foster, 2018). Such findings highlight the significance of self-efficacy, education, and exercise demonstration in promoting PA among older adults.

Nonetheless, given the multifactorial aetiology of ASCVD, aside from PA promotion, integrated CV interventions targeting multiple risk factors may provide a synthesis effect on ASCVD prevention. Han et al. (2018) investigated the impacts of CV health metrics on ASCVD incidence among 93987 Chinese adults. Approximately 17.4% of ASCVD, 16.7% of stroke and 18.0% of CHD could be prevented by attaining four lifestyle behaviours including regular PA, normal body mass index (BMI), smoking cessation and healthy diet. Thus, an integrated exercise and CV health education programme (HE programme) may provide additional benefits on ASCVD prevention targeting older adults. However, to date, related review examining the impacts of HE programmes on older adults in terms of PA level, ASCVD-related outcomes, and self-efficacy is scarce. Hence, a literature search was conducted from inception to January 13, 2021. Seven databases involving MEDLINE, CINAHL Complete, Science Direct, British Nursing Index, ProQuest, PsycINFO and PubMed were used. Additionally, manual searching and citation chaining were performed. Consequently, eight studies with a sum of 768 community-dwelling older adults were involved in the review. PA components of the included studies comprise either advice or exercise class. Details of search histories are enclosed in Appendices 1.

**Existing Research Gaps**

**Controversial programme impacts.** PA level was examined in three included studies, among which one involved physical advice (S. Yang et al., 2016) and two included exercise class (Park et al., 2011; Resnick et al., 2009). The one-group study by Resnick et al. (2009) revealed non-significant result. Meanwhile, two experimental studies revealed conflicting results (Park et al., 2011; S. Yang et al., 2016). Hence, current evidence is insufficient to draw a convincing inference.

ASCVD-related outcomes including blood pressure (BP), weight, waist circumference, BMI, blood glucose level and blood lipids were assessed. BP was examined in six studies, among which two involved physical advice (S. Yang et al., 2016; Murphy et al., 2015) and four included exercise class (Brokaw et al., 2015; Park et al., 2011; Resnick et al., 2009; Xu et al., 2015). Two one-group studies (Brokaw et al., 2015; Resnick et al., 2009) indicated significant SBP and DBP reductions in intervention groups. However, conflicting results were indicated among quasi-experimental studies and CCT (Park et al., 2011; S. Yang et al.,2016; Xu et al., 2015). Hence, its benefit on BP needs to be ascertained. Four studies examined the programme impact on BMI or weight respectively. A one-group study by Brokaw et al. (2015) and three quasi-experimental studies (Fox et al., 1996; S. Yang et al.,2016; Xu et al., 2015) revealed non-significant results on BMI. Meanwhile, the programme benefit on weight could not be confirmed as *p* values were mostly unreported (Amundson et al, 2009; Brokaw et al., 2015; Murphy et al., 2015). The programme impacts on waist circumference, blood glucose level and lipid level require further investigation as they were examined in few studies.

Further research is needed to confirm its positive impact on self-efficacy owing to limited supporting evidence (Park et al., 2011; S. Yang et al., 2016). Additionally, in the study by S. Yang et al. (2016), the effect on self-efficacy might not be generalisable as the evaluation tool mainly focused on hypertension control.

**Lack of empirical studies.** As only one included study is RCT while others are quasi-experimental or one-group studies, existing findings are relatively exploratory. Additionally, existing studies have limited sample size ranging from 8 to 41 (Amundson et al., 2009; Fox et al., 1996; Murphy et al., 2015; Park et al., 2011; Resnick et al., 2009; Xu et al., 2015). Furthermore, study transferability is limited as few were implemented in Asia (Park et al., 2011; S. Yang et al., 2016).

Programme impact may be affected by inconsistent intervention dosage. Notably, the attendance rates in most studies with exercise class are relatively poor, not to mention the lower attendance rate for exercise class compared with lecture (Amundson et al., 2009; Brokaw et al., 2015; Park et al., 2011; Resnick et al., 2009; Xu et al., 2015). One possible reason for suboptimal exercise adherence is the lack of self-efficacy among older adults (Rivera-Torres et al., 2019; Room et al., 2017) as few studies employed self-efficacy related theories in programme development. Another reason may be the inclusion of numerous exercise sessions in these programmes, in which older adults need to attend a minimum of two exercise sessions every week over a long duration. Hence, a booster intervention is needed to maintain the motivation among older adults while the HE programme needs to be simplified.

**Booster Intervention: Text Messaging**

Despite text messaging being rarely adopted in related studies, it has been adopted in other researches and achieved positive results. Text messaging was adopted in a 12-week exercise programme in older adults (55 to 70 years old) (Müller et al., 2016). The intervention group performed more exercises significantly than the control group. Additionally, participants expressed that text messaging was encouraging, motivating, and reminding. Likewise, Lilje et al. (2017) examined that effect of one-way text messages on reminding older adults with low back pain to perform exercises. Participants welcomed receiving text messages as they were useful, reminding, and timely. Moreover, Schwebel and Larimer (2018) reviewed the adoption of text messaging in healthcare services and revealed that it was reminding and enhanced medical compliance. As WHO (2015) recommends that health promotion messages delivered to older adults should be concise and positive, short message service (SMS) messaging can be considered a booster intervention to sustain their motivation across the study.

**Justification of the Proposed Research**

Current studies examining the effects of HE programmes on PA level, BP, BMI, weight, waist circumference, blood glucose, blood lipids and self-efficacy require further investigation. Study quality is limited to unmatched study design, limited sample size and transferability, varied intervention dosage and insufficient support of self-efficacy framework. Hence, further research in the form of theory-based, definitive RCT is needed.

A booster intervention is needed to maintain the motivation of older adults throughout the programme. A simplified HE programme, which includes the integration of one-time centred-based CV education accompanied by an educational booklet and the demonstration of an easy, home-based exercise inclines to be simple, cost-effective, and practical. Such innovation is increasingly imperative owing to pandemic lockdown, which makes the exercise which is easy and can be done at home a better PA option among older adults.

**Research Proposal**

**Aims**

The RCT aims at examining the effects of the HE programme for community-dwelling older adults at risk of ASCVD on PA level, ASCVD-related outcomes and exercise self-efficacy.

**Objectives**

Through implementing a HE programme on community-dwelling older adults at risk of ASCVD, compared with the control group,

1. the intervention group will achieve a significant increase in total PA score (PASE score) through by the end of 24 weeks (primary outcome);
2. the intervention group will attain significant improvement on ASCVD-related outcomes (including decreases in systolic and diastolic blood pressures, weight reduction, better BMI, reductions on waist circumference, blood glucose level and blood lipids, and increase in walking distance performed in the 2-MWT) through physiological evaluations and;
3. the intervention group will achieve a significant increase exercise self-efficacy score (SEE-C score).

**Theoretical Framework**

Self-Efficacy Theory was suggested by Bandura and Adams (1977). Self-efficacy is regarded as a person’s perception in his/her own capability of attaining something successfully (Brown et al., 2005). It is an essential factor of behavioural change as it affects a person’s coping effort, persistence, and decision in performing behaviours. Self-Efficacy Theory has been widely applied in researches about cardiac rehabilitation, self-management or health promotion programmes (Jeng & Braun, 1994; Peyman et al., 2020; Wu & Chang, 2014). Additionally, as aforementioned, self-efficacy is a motivator in the initiation, maintenance, and adherence of PA behaviours among older adults. Hence, incorporating Self-Efficacy Theory in the HE programme among older people with ASCVD risk appears to be beneficial.

**Theory application (Appendix 2).** Self-efficacy will be increased via enhancing its components including mastering experience, vicarious experience, verbal persuasion as well as emotional states (Brown et al., 2005).

Mastering experience will be strengthened if a person is provided technique, information, and belief in following lifestyle behaviours (Brown et al., 2005). It will be enhanced via conducting a HE session supported with exercise demonstration and group practice. Additionally, it will be reinforced via viewing exercise video, demonstration photos and healthy lifestyle information in the booklet and through delivery of educational points by SMS messaging.

Vicarious experience will be improved via observing peers’ successes (Brown et al., 2005). It will be enhanced via conducting group-based HE session, peer-sharing activity, and group exercise. Additionally, older adults will be invited to demonstrate exercise in the exercise video.

Verbal persuasion will be strengthened via encouraging a person that he/she will be able to master healthy lifestyle behaviours (Brown et al., 2005). During the HE session, encouragement will be provided to those who answer questions correctly. Additionally, encouraging words and exercise reminders will be delivered via SMS messages to maintain participants’ motivation towards performing PA.

Emotional states will be improved via evaluating a person’s levels of stress or worry on maintaining lifestyle behaviours and providing corresponding support (Brown et al., 2005). During the HE session, participants will be encouraged to share their barriers and opportunities towards performing PA. Meanwhile, common barriers and related coping strategies will be addressed via SMS messages. Additionally, an inquiry number will be provided in the booklet to provide participants’ additional support.

**Study design (Appendix 3)**

The pretest-posttest RCT is designed to be a parallel-group, two-arm, outcome assessor and participant blinded study with 1:1 allocation ratio. Following recruitment, informed consent and data collection at baseline, participants will be allocated randomly to either intervention or control group, in which the former will receive HE programme while the latter will receive usual care. Physiological evaluations, medical history-taking and self-administered questionnaires will be adopted for outcome assessment at baseline (T0), Week 12 (T1), Week 24 (T2) and Week 36 (T3).

**Participants**

**Inclusion criteria.** Community-dwelling adults are eligible if they fulfil the following inclusion criteria:

a) Chinese adults ≧60 years old;

b) Having at least one ASCVD risk factor (Appendix 4) (AHA, 2015; CDC, 2019; CDC, 2020a; WHO, 2011; WHO, 2017);

c) Pass the cardiovascular fitness evaluation (Complete the Get Active Questionnaire, without alarming resting HR (>100bpm) or BP (SBP>160mmHg, DBP>90mmHg), able to perform brisk walk (2-MWT);

d) Able to write and read Chinese, and communicate in Cantonese;

e) Possess a mobile phone and able to make use of the phone in reading SMS messages.

**Exclusion criteria.** a) Participants are excluded if they are visually impaired, hearing impaired, or suffer from cognitive, psychiatric, or muscular disorder as these conditions may limit the ability of comprehending educational materials, using mobile phone or performing PA;

b) Participants having a history of attending CV programme are excluded to prevent previous exposure to similar intervention;

c) As the study focuses on ASCVD prevention among community-dwelling adults, participants having a previous history of CHD or stroke are excluded.

**Settings**

Recruitment, data collection and intervention programme will be conducted in multiple elderly community centres in Macao.

**Interventions**

**Usual care.** At Week 1, control group will receive a group-based talk (60 to 70 minutes) on basic health issues (4 to 6 participants per group). Information leaflets, which are similar to the session content and are published by Macao Health Bureau, will be provided for home reference. A SMS reminder message on attending session will be sent one day before the talk. Information regarding PA and cardiovascular health education will not be provided during the talk. A 17-inch portable video player which contains a pre-recorded video regarding the placebo health education talk and a corresponding governmental leaflet will be delivered as reference. Aside from receiving SMS reminder messages on attending the talk and data collection, no other SMS messages will be given.

**HE programme (Appendix 5).**

***Programme development***. Previous studies highlight the importance of education and exercise demonstration in promoting PA among older adults. Meanwhile, current studies regarding HE programmes reveal that group education supported by booklet remains the mainstream of teaching. As suboptimal exercise adherence has been indicated in previous studies, a booster intervention in the form of SMS messaging and a simplified HE programme incline to be a better option. Hence, the HE programme comprises a HE session with exercise demonstration and group practice, a booklet, and a booster intervention via SMS messaging. As review findings suggest the incorporation of self-efficacy related framework in programme establishment, strategies for delivering the HE programme is designed with the support of Self-Efficacy Theory.

***Educational content.*** The exercise is developed according to international guidelines and relevant researches (Robinson et al., 2014; The Department of Health and Human Services, 2018; WHO, 2020). WHO (2020) and CDC (2018) recommended that older adults should engage in a minimum of either 150-minute aerobic PA with moderate intensity or 75-minute aerobic PA with vigorous intensity or a proportional combination of both every week. Additionally, the PA conducted is suggested to be multicomponent (strength training and functional balance). Meanwhile, review findings indicated that the exercises performed by elderly participants at risk of ASCVD, which primarily included walking, marching, stretching, dance steps, resistance exercise or Tai Chi, were mostly of moderate intensity and were normally well tolerated (Amundson et al., 2009; Brokaw et al., 2015; Fox et al., 1996; Resnick et al., 2009; S. Yang et al., 2016; Xu et al., 2015). Hence, the tailor-made exercise is moderate-intensity and involves an integration of balance training, aerobic and muscle-strengthening activities. Despite exercise class being mostly adopted in current studies, it was subject to an issue of suboptimal exercise adherence. Several systematic reviews and meta-analyses revealed that home-based exercise might be related to enhanced adherence, while it was also shown to be safe and effective in improving older adults’ physical outcomes in terms of muscle strength, balance, muscular endurance, and improved disability (Ashworth et al., 2005; Chaabene et al., 2021; Clegg et al., 2012; Mahjur & Norasteh, 2021). As such, the exercise would be designed to be performed at home or in group setting. Despite chair-based exercise being rarely employed in current studies, it was considered safe, simple, and easily implemented for the middle-aged, older adults and nursing home residents and it had been shown to have positive impacts on maintaining or improving their physical functions in two systematic reviews (Cordes et al., 2021; Klempel et al., 2021). Hence, the exercise comprises moderate-intensity exercises using a chair and a towel. To fulfil the WHO and CDC’s PA recommendations, the exercise duration lasts 25 minutes, with 5-minute warm-up, 15-minute exercises and 5-minute cool-down activities. Participants are encouraged to perform the exercise twice a day.

Educational contents of current HE programmes were primarily divided into two categories. Four studies included PA and general lifestyle modifications (Murphy et al., 2015; Park et al., 2011; Resnick et al., 2009; S. Yang et al., 2016) while others focused on PA and diet instructions (Amundson et al., 2009; Brokaw et al., 2015; Fox et al., 1996; Xu et al., 2015). Meanwhile, the reference source was relatively diverse and were mostly generated from international guidelines (such as the AHA’s Life Simple health guide, the Dietary Approaches to Stop Hypertension (DASH) programme, CDC’s National Diabetes Prevention Programme curriculum), regional guideline or literature review. To ensure the educational contents of the HE session to be evidence-based and in some cases more adapted to the local situation (All Asian studies focused on PA and general lifestyle modifications), the educational contents will focus on PA and general lifestyle modifications and they will be derived from international and regional guidelines on PA recommendation and ASCVD prevention (AHA, 2018a; CDC, 2019, 2020a; Department of Health and Human Services, 2018; the Joint Task Force for Guideline on the Assessment and Management of Cardiovascular Risk in China, 2019; WHO, 2010, 2020). While PA will be emphasised in the session, other educational contents will cover key elements of ASCVD prevention including smoking cessation, diet, and management on weight, BP, cholesterol, and blood glucose level.

Booklet content is basically coincident with the session content*.* Other than CV lifestyle modifications, it includes PA information on its advantages, exercise types, exercise intensity, demonstration pictures and a QR code for exercise video. Additionally, an inquiry number will be provided for answering participant concerns about the programme.

***Internal validity****.* Informational materials including exercise video, session PowerPoint slides, booklet and SMS messages will be previewed by an expert panel including seven healthcare professionals (two associate professors, one assistant professor, one physiotherapist, one cardiac nurse specialist, one cardiologist and one registered dietitian). The programme will be modified based on expert comments until its Content Validity Index reaches 0.8 (Rubio et al., 2003).

To ensure readability, the booklet will be designed with respect to the health literacy of older adults (CDC, 2020b). Additionally, informational materials will be previewed by three older adults at a maximum level of sixth grade (National Institutes of Health, 2013).

Principal investigator (PI) nurse will be responsible for preparing informational materials, conducting session and training, preparing progress report, delivering SMS messages, and answering participant’s phone inquiry if needed. To avoid subject contamination, session will be conducted in a private room and the two groups will receive their sessions at different times. Intervention group will receive a HE session conducted by PI nurse while control group will receive a talk conducted by community nurse if possible. To ensure standardised lecture delivery, a 45-minute training will be provided to lecture deliverer of the control group. PI nurse will explain details regarding lecture delivery. Differences in educational contents between control and intervention group will be highlighted. The remaining time will be used for inquiries. PowerPoint slides and leaflets will be provided for home reference. To ensure lecture quality, 10% of the intervention will be audio-recorded for quality control.

In most related studies, self-recording lifestyle patterns were encouraged (Amundson et al., 2009; Brokaw et al., 2015; Fox et al., 1996; Murphy et al., 2015; Resnick et al., 2009; Xu et al., 2015; Yang et al, 2016). Hence, to ensure intervention integrity, intervention group will be required to document their PA patterns (adherence on performing the tailor-made exercise, frequency of performing other exercises) in the exercise log every day. Additionally, they will be reminded to do the self-recording by weekly SMS messages. The exercise log will be collected at the end of 12 weeks.

***Programme safety.*** Although the tailor-made, chair-based exercise is considered relatively safe for older adults in previous studies, it is a moderate-intensity activity and hence it is needed to check older adults’ cardiorespiratory fitness before programme commencement to ensure exercise safety. The screening procedures included three phases. First, potential participants will complete a 4-item PA screening tool known as “Get Active Questionnaire” developed by the Canadian Society for Exercise Physiology (CSEP) in 2017. It is an acceptable tool which can be used in people with all ages and has been tested in older adult population (CSEP, 2021). The resting heart rate and BP of potential participants will then be assessed and those with an increased risk of adverse event during exercise (resting heart rate of more than 100 bpm or systolic BP of more than 160 mmHg or diastolic BP of more than 90 mmHg) will not be proceeded to the next phase unless they obtain the exercise approval from physicians (CSEP, 2021). As brisk walking is considered a moderate-intensity exercise, in the final phase, potential participants will take a cardiac endurance test known as two-minute walking test (2-MWT) to determine their aerobic capacity and balance capacity. For those who find themselves difficult to perform the walking test or those who experience extreme difficulty of breathing during the walking test will not be recruited into the study. In addition, the medical history of potential participants will be reviewed and those with previous history of myocardial infarction and CHD will be excluded from the study.

During the HE programme, precautions of performing PA will be addressed via lecture, booklet, and SMS messages. Participants will be encouraged to report the adverse events and express their concerns regarding exercise safety to the PI nurse via inquiry calls (with an inquiry number stated in the booklet).

Programme safety will also be assessed by measuring the number of adverse events throughout the study.

***Intervention procedures.*** The intervention will last 12 weeks. At Week 1, intervention group will receive a face-to face, group-based HE session (60-70 minutes) in elderly community centres (4 to 6 participants per group). Intervention group will receive a SMS reminder message one day before session. A tailor-made booklet will be provided for reference before session commencement. PI nurse will spend 30 minutes in teaching ASCVD prevention and multicomponent exercise. Participants will be invited to share their opinions about performing PA. Positive feedbacks will be given based on their sharing. Another 15 minutes will be spent in demonstrating exercise techniques. Finally, participants will be encouraged to perform the exercise together. These simple exercises are at moderate-intensity and involve an integration of balance training, aerobic and muscle-strengthening activities using a chair and a towel. To fulfil the PA recommendations suggested by the World Health Organisation (a minimum of either 150-minute aerobic PA with moderate intensity or 75-minute aerobic PA with vigorous intensity or a proportional combination of both every week), participants will be encouraged to perform these exercises twice a day, 25 minutes each time in which 5-minute warm-up and 5-minute cool down exercises will be included. To enhance the standardisation of intervention delivery, a tailor-made exercise video, in which a nurse and two elders take the lead in performing the aforementioned exercises, will be displayed during exercise demonstration. Additionally, to facilitate participants to revise the education materials and practice the exercises at home, each of them will receive a 17-inch portable video player which contains a pre-recorded lecture video and an exercise video following the talk. A telephone number will also be provided for the participants in case they have any question or worry regarding the programme. Intervention group will be required to document their PA patterns in the exercise log (enclosed in the booklet) every day.

Intervention group will then receive a booster intervention via SMS messaging. As comparable benefits were demonstrated between high-frequency and low-frequency messaging (Whittaker et al., 2019), it will be appropriate to adopt three messages per week to maintain participants’ motivation while minimising the annoyance of receiving excessive messages. Hence, from Week 1 to Week 12, a total of 36 SMS messages (one message on every Monday, Wednesday, and Friday) will be delivered in the daytime between 9am and 5pm with reference to participant selection. Message contents primarily cover key educational points, encouraging words and reminders of performing PA. At Week 7, participants will receive a SMS progress report regarding their interim evaluation result, accompanied with corresponding advice and positive feedback. Message structure is modified from the study by Müller et al. (2016), and will include greeting, instruction/encouragement section and closing.

**Data Collection**

For the consideration of data collection period, the systematic review by Peiris et al. (2021) indicated that the timing of outcome measurement among 11 included studies regarding lifestyle modification ranged from 12 to 144 weeks, with 24 weeks being mostly adopted in the effective studies. Hence, in this study, the outcomes will be measured at baseline and Week 24, with extra measures at Week 12 and Week 36 added to serve as the interim and follow-up evaluations respectively. Data collection will include four phases: T0 (baseline), T1 (Week 12), T2 (Week 24) and T3 (Week 36). Outcome assessors will have a 1-hour training regarding data collection procedure, physiological assessment, and ways of completing questionnaires. Additionally, they will receive training PowerPoint slides for reference.

**Baseline (T0).** Convenience sampling will be adopted as it is feasible in community setting due to economy and convenience. Recruitment method will be a combination of active and passive recruitment strategies. An A3-size recruitment poster will be displayed in the elderly centre in advance so that potential participants know about the study and interested ones can register their interest with centre staff. As direct contact with potential participants is relatively effective in primary care settings or among older adults (Chatters et al., 2018; Ngune et al., 2012), a centre staff familiar with the study will be arranged at the centre for programme inquiries. In case of having activities in which a large number of potential participants will be involved, the PI nurse will be present at the elderly centre to promote the HE programme.

Subsequently, registered potential participants will be approached by a research assistant face-to-face and their eligibility will be screened via completing a structured checklist while their cardiovascular fitness will be evaluated to ensure exercise safety. Eligible participants will be invited to participate in the study and sign a written informed consent after illustrating study details.

Baseline data (T0) on demographic details, PA level and exercise self-efficacy will be collected via completing a structured questionnaire. Participants will then receive physiological assessments. The evaluation results of BP and 2-MWT assessed at the screening stage will also be included as the baseline data. Following T0, participants will be allocated randomly to intervention or control group and receive corresponding interventions.

**Follow ups (T1, T2 and T3).** Participants will complete the same questionnaire and undergo physiological assessments at all follow-ups. To prevent contamination, different data collection timeslots will be arranged. To ensure participants to attend the follow ups, a reminder message will be sent to them one day before every follow up. Additionally, participants will receive a financial incentive of MOP100 supermarket cash coupon for their programme engagement at T3 (Week 36).

**Outcomes**

Despite PA level being reported to have positive effects on BP reduction and ASCVD prevention, older adults are the least physically active compared with other age groups. Hence, it is crucial to launch a HE programme to promote PA among older adults and lessen the ASCVD-related risks via enhancing their self-efficacy. In this research, examination on the effects of the HE programme on PA level, ASCVD-related outcomes and exercise self-efficacy will be evaluated, among which PA level at Week 24 is considered the primary outcome.

**Effects of HE programme.**

***PA level.*** Total PA level will be quantified with the Chinese version of Physical Activity Scale for the Elderly (PASE-C). The 12-item questionnaire assesses PA level of community-dwelling adults aged 65 or above via investigating leisure, physical, household, and work-associated activities in the last seven days (Washburn et al., 1993). The total score (from zero to 400 or above) is quantified based on frequency values and weights for these activities. The higher the score, the higher the level of PA. PASE-C had been previously used in Asian populations aged 60 or above (Kwan et al., 2020; Ng et al., 2020) and possessed satisfactory test-retest reliability (Intraclass correlation coefficient (ICC): 0.81) (Ngai et al., 2012).

To determine whether participants’ PA levels meet the recommended guideline, the Chinese version of International Physical Activity Questionnaire (IPAQ-C) (short form) will be adopted. The 7-item IPAQ-C (short form) evaluates PA of adults aged 15 to 69 in the last seven days via measuring duration and frequency for vigorous-intensity activities, moderate-intensity activities, and walking. Participants are classified into “inactive”, “minimally active” or “health enhancing physical activity (HEPA) active” based on their PA level. This questionnaire was widely used, cost-effective and possessed satisfactory reliability (ICC ranges from 0.81 to 0.89) and comparable psychometric properties with the long form (Craig et al., 2003; Deng et al., 2008; van Poppel et al., 2010).

***Exercise self-efficacy.*** Chinese version of SEE (SEE-C) (9-items) assesses participants’ self-efficacy to continue performing PA in case of encountering barriers. Participants give their ratings based on a 0 (not confident) -10 (very confident) format, with total score ranging from 0 to 90. The higher the score, the greater the exercise self-efficacy. SEE-C were assessed in Chinese populations (Lee et al., 2009; Wong et al., 2020a). It significantly predicted PA and had satisfactory internal consistency. Additionally, SEE-C has been adopted in a nurse-led lifestyle modification programme in Hong Kong (Wong et al., 2020b).

***ASCVD-related outcomes (BP, fasting blood glucose, fasting blood lipids ((low-density lipoprotein cholesterol, and high-density lipoprotein cholesterol, and triglycerides), weight, BMI, waist circumference, and 2-MWT).*** BP will be measured in sitting position, on the same side using calibrated, automatic BP monitor. Participants will receive a SMS message about precautions of BP measurement one day before data collection (AHA, 2018b). They will take a 5-minute rest prior BP measurement and 1-minute interval will be provided between each measurement (AHA, 2018b). Average of two measurements will be reported (Xu et al, 2015; S. Yang et al., 2016). BMI will be calculated with reference to height and weight. The height stated on participants’ identity cards will be recorded. Weight will be assessed using calibrated scale and the result will be corrected to the nearest 0.1 kg (S. Yang et al., 2016). Waist circumference will be measured two times in a standing position at the midpoint between the upper point of the iliac crest and the bottom of the last rib using a soft measuring tape. Average of two measurements will be reported and corrected to the nearest 0.1 cm. Blood glucose and blood lipids will be measured via obtaining finger-prick blood samples using an auto-analyser. Participants are required to fast 8 hours before obtaining blood samples.

2-MWT will be used to assess aerobic capacity and self-paced walking capacity via recording the distance covered at two minutes (Physiopedia, 2021). It was easy to implement and possessed satisfactory and comparable psychometric properties with 6-MWT, Timed up-and-go test and Berg Balance Scale among older adults (Chan and Pin, 2019; Connelly et al., 2009; Pin, 2014).

The evaluation results of blood pressure, heart rate, two-minute walk test, BMI, weight, and waist circumference assessed at the screening stage will be included as the baseline data. As the systematic review by Peiris et al. (2021) revealed that it took more than 3 months for lifestyle modification programmes integrated with unsupervised PA to cause significant impacts on fasting blood glucose, while another systematic review by Aucott et al. (2011) indicated that lifestyle modification programmes targeted at weight loss might have positive impacts on long-term lipid profile, these two outcomes will be both measured at baseline (T0), Week 24 (T2), and Week 36 (T3) only.

**Sample size**

Taking PA level as the primary outcome, with reference to a meta-analysis by Conn et al. (2008), the overall mean weighted effect size of patient education on PA level among 17147 patients with chronically diseases is 0.45. The sample size of the proposed study is calculated via G*Power calculator (Faul et al., 2007). For a two-tailed test, with the desired statistical power of 0.80 and significance level of 0.05, the estimated sample size is 158. An attrition rate of 20% is assumed. Hence, a total of 190 participants (95 participants per group) will be recruited in the study.

**Randomisation and Blinding**

Eligible participants will be allocated to either intervention or control group with 1:1 allocation ratio by computerized random number generator. The random allocation sequence will be generated by a research assistant not engaging in recruitment. Assignment results will be put in sequentially numbered, opaque, sealed envelopes (SNOSE) (Dettori, 2010; Polit & Beck, 2012). Research assistants will draw the envelope in sequence to determine a participant’s group allocation. To minimise performance bias, research assistants will not acknowledge participants their group assignment. Likewise, outcome assessors will be blinded to group assignment to restrain detection bias. Since PI nurse is responsible for conducting lectures, she is unlikely to be blinded.

**Statistical Methods**

The collected data will be analysed by SPSS version 26. Intention-to-treat analysis will be used to estimate the effect of the integrated exercise and cardiovascular health education programme. Demographic data will be analysed, with mean and standard deviation for continuous variables while percentage and frequency for categorical variables. Comparability between the participants in the experimental and control groups will be confirmed by independent t-test for continuous variables and chi-square test for categorical variables. Generalized Estimating Equations models will be applied to determine the effect of the programme on exercise self-efficacy, PA levels and ASCVD-related outcomes. P-value of less than 0.05 will be regarded as statistically significant.

**Ethical Considerations**

The study will be implemented following the ethical approval from the Human Subjects Ethics Sub-committee of the Hong Kong Polytechnic University. RCT registration will be applied. Permission of adopting questionnaires will be obtained from corresponding authors.

Eligible participants will be acknowledged about research safety and their rights on participations, withdrawals, and refusals in providing information. Additionally, they will be given research information sheets and sufficient time for raising questions. Research assistant will obtain written consents from those who agree to join the study.

To ensure confidentiality, all participant-related information, questionnaires, or consent forms will be kept in locked cabinets with limited access. Participant identities will be concealed via coded identification numbers. Meanwhile, databases will be password-protected and only research team has access right. Data will be primarily used in the study and will be disposed five years following study completion.

**Financial incentives.** Participants are required to show-up in the screening, lecture, and multiple data collections. Giving incentives is considered as a way of transportation allowance as well as showing appreciation to the participants for their effort and time spent in the study. Participants will receive MOP100 supermarket cash coupon at T3 (Week 36). Incentive details will be stated on the recruitment poster and information sheet.

**Discussion**

**Limitations**

Despite the HE programme being evidence-based and theory-driven, it has four limitations. First, this study will only evaluate the synthetic impacts of the HE programme on PA level, exercise self-efficacy and ASCVD outcomes. Second, as the programme is conducted in Cantonese and Chinese in consideration of time and economy, study applicability may be limited as foreign participants are excluded. Third, as participants need to undergo multiple data collection procedures, a higher absence rate may exist in the post-tests. Hence, a reminder message will be sent to remind participants the evaluation arrangement. Fourth, although several precautions have been made to prevent contamination, there is still a risk that participants may know other group’s allocated treatment as they are from the same centre.

**Implications**

**Advantages to research.** This study findings will provide clues to the effect of HE programme, which is theoretically underpinned with self-efficacy theory, in older adults at risk of ASCVD.

**Advantages to clinical practice.** As the healthcare burden of ASCVD is enormous and long-term, promoting HE programmes on older adults tends to be cost-effective. Additionally, the exercise designed for older adults, which is easy and safe to perform in any settings, is resource-saving and can be widely practicable in community settings. The study further provides clues for community healthcare professionals in determining the effective strategies targeting older adults, thereby improving the quality of community health education.

**Advantages to participants.** Current studies on ASCVD prevention seldom focus on older adults. The study designed for older adults helps to increase their awareness towards ASCVD prevention and builds up their self-efficacy on healthy lifestyle modification.

**Conclusion**

Despite older adults being increasingly exposed to the risk of ASCVD, they are often underrepresented in ASCVD prevention programmes. Review findings suggest that further empirical, theory-based RCT is needed to explore the synthesis impact of integrated exercise and CV health education programmes (HE programmes) on PA level, ASCVD outcomes and exercise self-efficacy for community-dwelling older adults at risk of ASCVD. This study will examine the programme effects by investigating whether a simplified HE programme supported by SMS messaging will be a better approach compared with usual care. Study findings will provide new insight on the effective strategy used in the ASCVD prevention targeting older adults.

**References**

American Heart Association. (2018b). *Blood pressure measurement instructions*. Retrieved February 11, 2021, from https://www.heart.org/-/media/files/health-topics/high-blood-pressure/how_to_measure_your_blood_pressure_letter_size.pdf

American Heart Association. (2015). *Family history and heart disease, stroke*. Retrieved October 13, 2020, from https://www.heart.org/en/health-topics/consumer-healthcare/what-is-cardiovascular-disease/family-history-and-heart-disease-stroke

American Heart Association. (2018a). *My life check | Life's simple 7*. Retrieved October 15, 2020, from https://www.heart.org/en/healthy-living/healthy-lifestyle/my-life-check--lifes-simple-7

Amundson, H. A., Butcher, M. K., Gohdes, D., Hall, T. O., Harwell, T. S., Helgerson, S. D., Vanderwood, K. K., & Montana Cardiovascular Disease and Diabetes Prevention Program Workgroup (2009). Translating the diabetes prevention program into practice in the general community: findings from the Montana Cardiovascular Disease and Diabetes Prevention Program. *The Diabetes Educator, 35*(2), 209-223. https://doi.org/10.1177/0145721709333269

Armijo-Olivo, S., Stiles, C. R., Hagen, N. A., Biondo, P. D., & Cummings, G. G. (2012). Assessment of study quality for systematic reviews: A comparison of the Cochrane Collaboration Risk of Bias Tool and the Effective Public Health Practice Project Quality Assessment Tool: Methodological research. *Journal of Evaluation in Clinical Practice, 18*(1), 12–18. doi:10.1111/j.1365-2753.2010.01516.x

ARUP Consult. (2020). *Atherosclerotic Cardiovascular Disease Risk Markers*. Retrieved August 28, 2020, from <https://arupconsult.com/content/cardiovascular-disease-traditional-risk-markers>

Ashworth, N. L., Chad, K. E., Harrison, E. L., Reeder, B. A., & Marshall, S. C. (2005). Home versus center based physical activity programs in older adults. *The Cochrane Database of Systematic Reviews, 2005*(1), CD004017. <https://doi.org/10.1002/14651858.CD004017.pub2>

Aucott, L., Gray, D., Rothnie, H., Thapa, M., & Waweru, C. (2011). Effects of lifestyle interventions and long-term weight loss on lipid outcomes - a systematic review. *Obesity Reviews : an official journal of the International Association for the Study of Obesity, 12*(5), e412–e425. https://doi.org/10.1111/j.1467-789X.2010.00819.x

Bandura, A., & Adams, N. E. (1977). Analysis of self-efficacy theory of behavioral change. *Cognitive Therapy and Research, 1*(4), 287-310.

Barry, A. R., O'Neill, D. E., & Graham, M. M. (2016). Primary prevention of cardiovascular disease in older adults. *The Canadian Journal of Cardiology, 32*(9), 1074–1081. doi: 10.1016/j.cjca.2016.01.032

Benjamin, E. J., Muntner, P., Alonso, A., Bittencourt, M. S., Callaway, C. W., Carson, A. P., Chamberlain, A. M., Chang, A. R., Cheng, S., Das, S. R., Delling, F. N., Djousse, L., Elkind, M. S. V., Ferguson, J. F., Fornage, M., Jordan, L. C., Khan, S. S., Kissela, B. M., Knutson, K. L.,…Virani, S. S. (2019). Heart disease and stroke statistics-2019 update: A report from the American Heart Association. *Circulation, 139*, e56-e528. doi:10.1161/CIR.0000000000000659

Bethancourt, H. J., Rosenberg, D. E., Beatty, T., & Arterburn, D. E. (2014). Barriers to and facilitators of physical activity program use among older adults. *Clinical Medicine & Research, 12*(1-2), 10–20. doi:10.3121/cmr.2013.1171

Brokaw, S. M., Carpenedo, D., Campbell, P., Butcher, M. K., Furshong, G., Helgerson, S. D., Harwell, S. D. (2015). Effectiveness of an adapted diabetes prevention program lifestyle intervention in older and younger adults. *Journal of the American Geriatrics Society, 63*(6), 1067-1074.

Brown, L. J., Malouff, J. M., & Schutte, N. S. (2005). *Chapter 2 Self-efficacy theory.* Retrieved October 1, 2020, from http://samples.jbpub.com/9781449689742/Chapter2.pdf

Bull, F. C., Armstrong, T. P., Dixon, T., Ham, R., Neiman, A., & Pratt, M. (2004). *Physical inactivity.* Retrieved January 30, 2021, from https://www.who.int/publications/cra/chapters/volume1/0729-0882.pdf

Canadian Society for Exercise Physiology. (2021). Pre-screening for physical activity: Get active questionnaire. Retrieved 20 May, 2021, from https://store.csep.ca/pages/getactivequestionnaire

Cavill, N. A., & Foster C. E. M. (2018). Enablers and barriers to older people’s participation in strength and balance activities: A review of reviews. *Journal of Frailty, Sarcopenia and Falls, 3*(2), 105-113. doi: 10.22540/JFSF-03-105

Centers for Disease Control and Prevention. (2020a). *Heart disease and stroke*. Retrieved October 13, 2020, from https://www.cdc.gov/tobacco/basic_information/health_effects/heart_disease/index.htm

Centers for Disease Control and Prevention. (2019). *Know your risk for heart disease*. Retrieved October 13, 2020, from https://www.cdc.gov/heartdisease/risk_factors.htm

Centers for Disease Control and Prevention. (2020b). *Understanding & use of health information*. Retrieved October 17, 2020, from https://www.cdc.gov/healthliteracy/developmaterials/audiences/olderadults/steps.html/

Chaabene, H., Prieske, O., Herz, M., Moran, J., Höhne, J., Kliegl, R., Ramirez-Campillo, R., Behm, D. G., Hortobágyi, T., & Granacher, U. (2021). Home-based exercise programmes improve physical fitness of healthy older adults: A PRISMA-compliant systematic review and meta-analysis with relevance for COVID-19. *Ageing Research Reviews, 67*, 101265. https://doi.org/10.1016/j.arr.2021.101265

Chan, W. L. S., & Pin, T. W. (2019). Reliability, validity and minimal detectable change of 2-minute walk test, 6-minute walk test and 10-meter walk test in frail older adults with dementia. *Experimental Gerontology, 115*, 9–18. https://doi.org/10.1016/j.exger.2018.11.001

Chatters, R., Newbould, L., Sprange, K., Hind, D., Mountain, G., Shortland, K., Powell, L., Gossage-Worrall, R., Chater, T., Keetharuth, A., Lee, E., & Woods, B. (2018). Recruitment of older adults to three preventative lifestyle improvement studies. *Trials, 19*(1), 121. https://doi.org/10.1186/s13063-018-2482-1

Clegg, A. P., Barber, S. E., Young, J. B., Forster, A., & Iliffe, S. J. (2012). Do home-based exercise interventions improve outcomes for frail older people? Findings from a systematic review. *Reviews in Clinical Gerontology, 22*(1), 68–78. https://doi.org/10.1017/S0959259811000165

Conn, V. S., Hafdahl, A. R., Brown, S. A., & Brown, L. M. (2008). Meta-analysis of patient education interventions to increase physical activity among chronically ill adults. *Patient Education and Counseling, 70*(2), 157–172. https://doi.org/10.1016/j.pec.2007.10.004

Connelly, D. M., Thomas, B. K., Cliffe, S. J., Perry, W. M., & Smith, R. E. (2009). Clinical utility of the 2-minute walk test for older adults living in long-term care. *Physiotherapy Canada, 61*(2), 78–87. https://doi.org/10.3138/physio.61.2.78

Cordes, T., Schoene, D., Kemmler, W., & Wollesen, B. (2021). Chair-based exercise interventions for nursing home residents: A systematic review. *Journal of the American Medical Directors Association, 22*(4), 733–740. https://doi.org/10.1016/j.jamda.2020.09.042

Craig, C. L., Marshall, A. L., Sjöström, M., Bauman, A. E., Booth, M. L., Ainsworth, B. E., Pratt, M., Ekelund, U., Yngve, A., Sallis, J. F., & Oja, P. (2003). International Physical Activity Questionnaire: 12-country reliability and validity. *Medicine and Science in Sports and Exercise, 35*(8), 1381–1395. https://doi.org/10.1249/01.MSS.0000078924.61453.FB

Deng, H. B., Macfarlane, D. J., Thomas, G. N., Lao, X. Q., Jiang, C. Q., Cheng, K. K., & Lam, T. H. (2008). Reliability and validity of the IPAQ-Chinese: The Guangzhou Biobank Cohort study. *Medicine and Science in Sports and Exercise, 40*(2), 303–307. doi:10.1249/mss.0b013e31815b0db5

Dettori, J. (2010). The random allocation process: Two things you need to know. *Evidence-based Spine-care Journal, 1*(3), 7–9. doi:10.1055/s-0030-1267062

Faul, F., Erdfelder, E., Lang, A. G., & Buchner, A. (2007). G*Power 3: a flexible statistical power analysis program for the social, behavioral, and biomedical sciences. *Behavior Research Methods, 39*(2), 175–191. https://doi.org/10.3758/bf03193146

Fox, A. A., Thompson, J. L., Butterfield, G. E., Gylfadottir, U., Moynihan, S., & Spiller, G. (1996). Effects of diet and exercise on common cardiovascular disease risk factors in moderately obese older women. *The American Journal of Clinical Nutrition, 63*(2), 225–233. https://doi.org/10.1093/ajcn/63.2.225

Han, C., Liu, F., Yang, X., Chen, J., Li, J., Cao, J., Li, Y., Shen, C., Yu, L., Liu, Z., Wu, X., Zhao, L., Hu, D., Lu, X., Wu, X., & Gu, D. (2018). Ideal cardiovascular health and incidence of atherosclerotic cardiovascular disease among Chinese adults: The China-PAR project. *Science China Life Sciences, 61*, 504–514. doi:10.1007/s11427-018-9281-6

Jeng, C., & Braun, L. T. (1994). Bandura's self-efficacy theory: A guide for cardiac rehabilitation nursing practice. *Journal of Holistic Nursing, 12*(4), 425–436. doi:10.1177/089801019401200411

Klempel, N., Blackburn, N. E., McMullan, I. L., Wilson, J. J., Smith, L., Cunningham, C., O'Sullivan, R., Caserotti, P., & Tully, M. A. (2021). The effect of chair-based exercise on physical function in older adults: A systematic review and meta-analysis. *International Journal of Environmental Research and Public Health, 18*(4), 1902. https://doi.org/10.3390/ijerph18041902

Konrat, C., Boutron, I., Trinquart, L., Auleley, G., Ricordeau, P., & Ravaud, P. (2012). Underrepresentation of elderly people in randomised controlled trials. The example of trials of 4 widely prescribed drugs. *PloS one, 7*(3), e33559.

Kwan, R. Y., Lee, D., Lee, P. H., Tse, M., Cheung, D. S., Thiamwong, L., & Choi, K. S. (2020). Effects of an mHealth brisk walking intervention on increasing physical activity in older people with cognitive frailty: Pilot randomized controlled trial. *JMIR MHealth and UHealth, 8*(7), e16596. https://doi.org/10.2196/16596

Lee, L. L., Perng, S. J., Ho, C. C., Hsu, H. M., Lau, S. C., & Arthur, A. (2009). A preliminary reliability and validity study of the Chinese version of the self-efficacy for exercise scale for older adults. *International Journal of Nursing Studies, 46*(2), 230–238. doi:10.1016/j.ijnurstu.2008.09.003

Lilje, S. C., Olander, E., Berglund, J., Skillgate, E., & Anderberg, P. (2017). Experiences of older adults with mobile phone text messaging as reminders of home exercises after specialized manual therapy for recurrent low back pain: A qualitative study. *JMIR Mhealth and Uhealth, 5*(3), e39. doi:10.2196/mhealth.7184

Macao Association for Internet Research (2019). *Internet Usage Trends in Macao 2019*. Macao: MAIR

Mahjur, M., & Norasteh, A. A. (2021). The effect of unsupervised home-based exercise training on physical functioning outcomes in older adults: A systematic review and meta-analysis of randomized controlled trials. *Biological Research for Nursing,* 1099800421989439. Advance online publication. https://doi.org/10.1177/1099800421989439

Marcus, B. H., Dubbert, P. M., Forsyth, L. H., McKenzie, T. L., Stone, E. J., Dunn, A. L., & Blair, S. N. (2000). Physical activity behavior change: Issues in adoption and maintenance. *Health Psychology, 19*(1S), 32–41. doi:10.1037/0278-6133.19.suppl1.32

McAuley, E., Jerome, G. J., Marquez, D. X., Elavsky, S., & Blissmer, B. (2003). Exercise self-efficacy in older adults: Social, affective, and behavioral influences. *Annals of Behavioral Medicine, 25*(1), 1–7. doi: 10.1207/S15324796ABM2501_01

McAuley, E., Morris, K. S., Motl, R. W., Hu, L., Konopack, J. F., & Elavsky, S. (2007). Long-term follow-up of physical activity behavior in older adults. *Health Psychology, 26*(3), 375–380. doi: 10.1037/0278-6133.26.3.375

McAuley, E., Szabo, A., Gothe, N., Olson, E. A. (2011). Self-efficacy: Implications for physical activity, function, and functional limitations in older adults. *American Journal of Lifestyle Medicine, 5*(4), 10.1177/1559827610392704. doi:10.1177/1559827610392704

McMaster University, School of Nursing. (1999). *Effective public health practice project.* Retrieved January 10, 2021, from https://merst.ca/ephpp/

Moher, D., Hopewell, S., Schulz, K. F., Montori, V., Gøtzsche, P. C., Devereaux, P. J., Elbourne, D., Egger, M., & Altman, D. G. (2010). CONSORT 2010 explanation and elaboration: updated guidelines for reporting parallel group randomised trials. *BMJ (Clinical research ed.), 340*, c869. doi:10.1136/bmj.c869

Mozaffarian, D., Benjamin, E. J., Go, A. S., Arnett, D. K., Blaha, M. J., Cushman, M., Das, S. R., de Ferranti, S., Després, J, Fullerton, H. J., Howard, V. J., Huffman, M. D., Isasi, C. R., Jiménez, M. C., Judd, S. E., Kissela, B. M., Lichtman, J. H., Lisabeth, L. D., Liu, S.,…, Turner, M. B. (2016). Heart disease and stroke statistics—2016 update: A report from the American Heart Association. *Circulation, 133*(4), e38-e360.

Müller, A. M., Khoo, S., & Morris, T. (2016). Text messaging for exercise promotion in older adults from an upper-middle-income country: Randomized controlled trial. *Journal of Medical Internet Research, 18*(1), e5. doi:10.2196/jmir.5235

Murphy, M. P., Coke, L., Staffileno, B. A., Robinson, J. D., & Tillotson, R. (2015). Improving cardiovascular health of underserved populations in the community with Life’s Simple 7. *Journal of the American Association of Nurse Practitioners, 27*, 615-623. doi: 10.1002/2327-6924.12231

National Institutes of Health. (2013). *How to write easy-to-read health materials*. Retrieved October 20, 2020, from http://www.nlm.nih.gov/medlineplus/etr.html

Ng, L. P., Koh, Y., & Tan, N. C. (2020). Physical activity and sedentary behaviour of ambulatory older adults in a developed Asian community: A cross-sectional study. *Singapore Medical Journal, 61*(5), 266–271. https://doi.org/10.11622/smedj.2020022

Ngai, S. P., Cheung, R. T., Lam, P. L., Chiu, J. K., & Fung, E. Y. (2012). Validation and reliability of the Physical Activity Scale for the Elderly in Chinese population. *Journal of Rehabilitation Medicine, 44*(5), 462–465. <https://doi.org/10.2340/16501977-0953>

Ngune, I., Jiwa, M., Dadich, A., Lotriet, J., & Sriram, D. (2012). Effective recruitment strategies in primary care research: A systematic review. *Quality in Primary Care, 20*(2), 115–123.

Norman, G. (2010). Likert scales, levels of measurement and the “laws” of statistics. *Advances in Health Sciences Education, 15*(5), 625-632.

Park, Y. H., Song, M., Cho, B. L., Lim, J. Y., Song, W., & Kim, S. H. (2011). The effects of an integrated health education and exercise program in community-dwelling older adults with hypertension: A randomized controlled trial. *Patient Education and Counseling, 82*(1), 133–137. doi:10.1016/j.pec.2010.04.002

Peiris, C. L., van Namen, M., & O'Donoghue, G. (2021). Education-based, lifestyle intervention programs with unsupervised exercise improve outcomes in adults with metabolic syndrome. A systematic review and meta-analysis. *Reviews in Endocrine & Metabolic Disorders,* 22(4), 877–890. https://doi.org/10.1007/s11154-021-09644-2

Peyman, N., Shahedi, F., Abdollahi, M., Doosti, H., & Zadehahmad, Z. (2020). Impact of self-efficacy strategies education on self-care behaviors among heart failure patients. *The Journal of Tehran Heart Center, 15*(1), 6–11.

Physiopedia. (2021). *2 minute walk test*. Retrieved February 15, 2021, from https://www.physio-pedia.com/2_Minute_Walk_Test

Pin, T. W. (2014). Psychometric properties of 2-minute walk test: A systematic review. *Archives of Physical Medicine and Rehabilitation, 95*(9), 1759–1775. https://doi.org/10.1016/j.apmr.2014.03.034

Polit, D. F., & Beck, C. T. (2012). *Nursing research: Generating and assessing evidence for nursing practice* (9th ed.). Philadelphia, PA: Wolters Kluwer Health/Lippincott Williams & Wilkins.

Preston, C. C., & Colman, A. M. (2000). Optimal number of response categories in rating scales: Reliability, validity, discriminating power, and respondent preferences. *Acta Psychologica, 104*(1), 1-15.

Resnick, B., Shaughnessy, M., Galik, E., Scheve, A., Fitten, R., Morrison, T., Michael, K., & Agness, C. (2009). Pilot testing of the PRAISEDD intervention among African American and low-income older adults. *The Journal of Cardiovascular Nursing, 24*(5), 352–361. https://doi.org/10.1097/JCN.0b013e3181ac0301

Rivera-Torres, S., Fahey, T. D., & Rivera, M. A. (2019). Adherence to exercise programs in older adults: Informative report. *Gerontology & Geriatric Medicine, 5*, 2333721418823604. doi:10.1177/2333721418823604

Robinson, K. R., Leighton, P., Logan, P., Gordon, A. L., Anthony, K., Harwood, R. H., Gladman, J. R., & Masud, T. (2014). Developing the principles of chair based exercise for older people: A modified Delphi study. *BMC Geriatrics, 14*, 65. https://doi.org/10.1186/1471-2318-14-65

Room, J., Hannink, E., Dawes, H., & Barker, K. (2017). What interventions are used to improve exercise adherence in older people and what behavioural techniques are they based on? A systematic review. *BMJ Open, 7*(12), e019221. doi:10.1136/bmjopen-2017-019221

Rubio, D. M., Berg-Weger, M., Tebb, S. S., Lee, E. S., & Rauch, S. (2003). Objectifying content validity: Conducting a content validity study in social work research. *Social Work Research, 27*(2), 94–104. https://doi.org/10.1093/swr/27.2.94

Sardar, M. R., Badri, M., Prince, C. T., Seltzer, J., & Kowey, P. R. (2014). Underrepresentation of women, elderly patients, and racial minorities in the randomized trials used for cardiovascular guidelines. *JAMA Internal Medicine, 174*(11), 1868–1870. doi: 10.1001/jamainternmed.2014.4758

Schwebel, F. J., & Larimer, M. E. (2018). Using text message reminders in health care services: A narrative literature review. *Internet Interventions, 13*, 82–104. doi:10.1016/j.invent.2018.06.002

Sun, F., Norman, I. J., & While, A. E. (2013). Physical activity in older people: A systematic review. *BMC Public Health, 13*, 449. doi: 10.1186/1471-2458-13-449

Sullivan, G. M., & Artino, A. R. (2013). Analyzing and interpreting data from Likert-type scales. *Journal of Graduate Medical Education, 5*(4), 541-542.

The Chinese University of Hong Kong Jockey Club Institute of Ageing. (2020). *AgeWatch Index for Hong Kong: Topical report on capability*. Retrieved August 24, 2020, from https://www.jcafc.hk/uploads/docs/Topical-Report-Capability_final.pdf

The Department of Health and Human Services. (2018). *The physical activity guidelines for Americans* (2nd ed.). Retrieved February 15, 2021, from https://health.gov/sites/default/files/2019-09/Physical_Activity_Guidelines_2nd_edition.pdf

The Joint Task Force for Guideline on the Assessment and Management of Cardiovascular Risk in China. (2019). Guideline on the assessment and management of cardiovascular risk in China. *Chinese Circulation Journal, 34*(1), 4-28.

Thomas, B. H., Ciliska, D., Dobbins, M., & Micucci, S. (2004). A process for systematically reviewing the literature: Providing the research evidence for public health nursing interventions. *Worldviews on Evidence-Based Nursing, 1*(3), 176–184. doi:10.1111/j.1524-475X.2004.04006.x

United Nations, Department of Economic and Social Affairs, Population Division. (2017). *World population ageing 2017-Highlights (ST/ESA/SER.A/397)*. Retrieved August 24, 2020, from https://www.un.org/en/development/desa/population/publications/pdf/ageing/WPA2017_Highlights.pdf

van Poppel, M. N., Chinapaw, M. J., Mokkink, L. B., van Mechelen, W., & Terwee, C. B. (2010). Physical activity questionnaires for adults: A systematic review of measurement properties. *Sports Medicine, 40*(7), 565–600. doi:10.2165/11531930-000000000-00000

Warburton, D. E., Nicol, C. W., & Bredin, S. S. (2006). Health benefits of physical activity: The evidence. *Canadian Medical Association Journal, 174*(6), 801–809. doi:10.1503/cmaj.051351

Washburn, R. A., Smith, K. W., Jette, A. M., & Janney, C. A. (1993). The Physical Activity Scale for the Elderly (PASE): Development and evaluation. *Journal of Clinical Epidemiology, 46*(2), 153–162. https://doi.org/10.1016/0895-4356(93)90053-4

White, S. M., Wójcicki, T. R., & McAuley, E. (2012). Social cognitive influences on physical activity behavior in middle-aged and older adults. *The Journals of Gerontology. Series B, Psychological Sciences and Social Sciences, 67*(1), 18–26. doi:10.1093/geronb/gbr064

Whittaker, R., McRobbie, H., Bullen, C., Rodgers, A., Gu, Y., & Dobson, R. (2019). Mobile phone text messaging and app-based interventions for smoking cessation. *The Cochrane database of Systematic Reviews, 10*(10), CD006611. Advance online publication. https://doi.org/10.1002/14651858.CD006611.pub5

Wong, E. M. L., Leung, D. Y. P., Sit, J. W. H., Chan, A. W. K., & Chair, S. Y. (2020a). Prospective validation of the Chinese Version of the Self-Efficacy for Exercise Scale among middle-aged patients with coronary heart disease. *Rehabilitation Nursing, 45*(2), 74–79. doi:10.1097/RNJ.0000000000000156

Wong, E. M. L., Leung, D. Y. P., Wang, Q., & Leung, A. Y. M. (2020b). A nurse-led lifestyle intervention using mobile application versus booklet for adults with metabolic syndrome-Protocol for a randomized controlled trial. *Journal of Advanced Nursing, 76*(1), 364–372. doi:10.1111/jan.14241

World Health Organization. (2018). *Ageing and health.* Retrieved May 15, 2021, from <https://www.who.int/news-room/fact-sheets/detail/ageing-and-health>

World Health Organization. (2017). *Cardiovascular diseases (CVDs)*. Retrieved January 02, 2021, from https://www.who.int/en/news-room/fact-sheets/detail/cardiovascular-diseases-(cvds)

World Health Organization. (2011). *Global atlas on cardiovascular disease prevention and control*. Retrieved October 10, 2020, from https://apps.who.int/iris/rest/bitstreams/53236/retrieve

World Health Organization. (2015). *Health promotion for older people: Not business as usual.* Retrieved February 01, 2021, from <https://www.who.int/ageing/features/health-promotion/en/>

World Health Organization. (2010). *Package of essential noncommunicable (PEN) disease interventions for primary health care in low-resource settings*. Retrieved October 15, 2020, from https://www.who.int/activities/integrated-management-of-ncds

World Health Organization. (2020). *Physical activity.* Retrieved January 20, 2021, from https://www.who.int/news-room/fact-sheets/detail/physical-activity

World Health Organization. (2019). *WHO cardiovascular disease risk laboratory-based charts.* Retrieved January 10, 2021, from https://www.who.int/docs/default-source/ncds/cvd-risk-laboratory-based-charts.pdf?sfvrsn=e2943d98_2

World Health Organization. (2020). *WHO guidelines on physical activity and sedentary behaviour.* Retrieved February 10, 2021, from https://www.who.int/publications/i/item/9789240015128

Wu, C. J., & Chang, A. M. (2014). Application of a theoretical framework to foster a cardiac-diabetes self-management programme. *International Nursing Review, 61*(3), 336–343. doi:10.1111/inr.12104

Xu, F., Letendre, J., Bekke, J., Beebe, N., Mahler, L., Lofgren, I. E., & Delmonico, M. J. (2015). Impact of a program of Tai Chi plus behaviorally based dietary weight loss on physical functioning and coronary heart disease risk factors: A community-based study in obese older women. *Journal of Nutrition in Gerontology and Geriatrics, 34*(1), 50-65.

Yang, S. O., Kim, S. J., & Lee, S. H. (2016). Effects of a South Korean community-based cardiovascular disease prevention program for low-income elderly with hypertension. *Journal of Community Health Nursing, 33*(3), 154–167. https://doi.org/10.1080/07370016.2016.1191872

Zhao, D., Liu, J., Wang, M., Zhang, X., & Zhou, M. (2019). Epidemiology of cardiovascular disease in China: Current features and implications. *Nature Reviews Cardiology, 16*, 203–212.

**Appendix 1a**

*Systematic Search Histories via 7 Databases*

| Databases | Syntax | Limits | No. of Articles |
| --- | --- | --- | --- |
| PubMed | ((((older adults[Title/Abstract] OR elderly[Title/Abstract] OR geriatric*[Title/Abstract] OR aging[Title/Abstract] OR senior*[Title/Abstract] OR older people[Title/Abstract] OR aged[Title/Abstract]) AND (community-dwelling[Title/Abstract] OR community setting[Title/Abstract] OR community-based[Title/Abstract] OR community[Title/Abstract])) AND (CVD risk*[Title/Abstract] OR ASCVD risk*[Title/Abstract] OR atherosclerotic cardiovascular disease risk*[Title/Abstract] OR stroke risk*[Title/Abstract] OR myocardial infarction risk*[Title/Abstract] OR coronary heart disease risk*[Title/Abstract] OR CHD risk*[Title/Abstract] OR cardiac risk*[Title/Abstract])) AND (health promotion[Title/Abstract] OR health campaign[Title/Abstract] OR wellness program*[Title/Abstract] OR education*[Title/Abstract] OR program*[Title/Abstract] OR prevention[Title/Abstract])) AND (self efficacy[Title/Abstract] OR self-efficacy[Title/Abstract] OR ASCVD risk[Title/Abstract] OR CVD risk[Title/Abstract] OR Lifestyle[Title/Abstract] OR lifestyle change*[Title/Abstract] OR lifestyle modification[Title/Abstract] OR lifestyle choice*[Title/Abstract] OR risk modification*[Title/Abstract] OR risk reduction[Title/Abstract] OR risk reduction behavio*[Title/Abstract] OR health promotion behavio*[Title/Abstract]) | English, search by title/abstract | 142 |
| MEDLINE, CINAHL Complete | S1: AB older adults OR elderly OR geriatric* OR aging OR senior* OR older people OR aged  S2: AB community-dwelling OR community setting OR community-based OR community  S3: AB CVD risk* OR ASCVD risk* OR atherosclerotic cardiovascular disease risk* OR stroke risk* OR myocardial infarction risk* OR coronary heart disease risk* OR CHD risk* OR cardiac risk*  S4: AB health promotion OR health campaign OR wellness program* OR education* OR program* OR prevention  S5: AB self efficacy OR self-efficacy OR ASCVD risk OR CVD risk OR Lifestyle OR lifestyle change* OR lifestyle modification OR lifestyle choice* OR risk modification* OR risk reduction OR risk reduction behavio* OR health promotion behavio*  S6: S1 AND S2 AND S3 AND S4 AND S5 | Search by abstract | 336  EXACT 249  DUP  87 |
| Science Direct | (older) AND (community) AND (cardiovascular disease risk OR cardiac risk) AND (promotion OR prevention) AND (self efficacy OR ASCVD risk OR lifestyle) | Search by title, abstract or author-specified keywords, by research article | 14 |
| British Nursing Index, ProQuest Dissertations & Theses A&I,  APA PsycINFO | ab(older adults OR elderly OR geriatric* OR aging OR senior* OR older people OR aged) AND ab(community-dwelling OR community setting OR community-based OR community) AND ab(CVD risk* OR ASCVD risk* OR atherosclerotic cardiovascular disease risk* OR cardiac risk* OR CHD risk* OR coronary heart disease risk* OR myocardial infarction risk* OR stroke risk*) AND ab(health promotion OR health campaign OR wellness program* OR education* OR program* OR prevention) AND ab(self efficacy OR self-efficacy OR ASCVD risk OR Lifestyle OR lifestyle change* OR lifestyle modification OR lifestyle choice* OR risk modification* OR risk reduction OR risk reduction behavio* OR health promotion behavio*) | By Abstract, English | 100 |
|  |  | Total | 592 |

**Appendix 1b**

*Flow Diagram of Literature Search (PRISMA 2009 Flow Diagram)*

Additional records identified through manual searching and citation chaining
(n=31)

Records identified through database searching (CINAHL Complete, MEDLINE, British Nursing Index, PsycINFO, ProQuest, Science Direct, PubMed)
(n=592)

## Identification

Records after duplicates removed

(n=253)

## Screening

Records screened
(n=370)

Records excluded (n= 267)

Full-text articles excluded, with reasons (n=95)

1. Unmatched participants (n=53)
2. Irrelevant (n=40)
3. Protocols (n=2)

Full-text articles assessed for eligibility

(n=103)

**Eligibility**

Studies included in qualitative synthesis
(n=8)

**Included**

**Appendix 2**

*Application of Self-Efficacy Theory to the HE Programme*

| **Personal factors** |  | **Intervention components** |  | **Sources of self-efficacy** | | | | | | |  | **Outcomes** |
| --- | --- | --- | --- | --- | --- | --- | --- | --- | --- | --- | --- | --- |
|  |  |  |  |  | | | | | | |  |  |
| - Age - Gender - Existing knowledge - Education level - Atherosclerotic risks |  | - Health educational session: Group-based lifestyle modification session with exercise demonstration |  |  | | Mastery experience | | |  | |  |  |
|  |  |  |  |  |  |  | | |  |  |  |  |
|  |  |  |  |  |  |  | | |  |  |  |  |
|  |  |  |  |  | Vicarious experience | | | | |  |  |  |
|  |  |  |  |  |  |  |  |  |  |  |  | - Self-efficacy - Physical level - Outcomes related to atherosclerotic cardiovascular disease |
|  |  | - Educational booklet (lifestyle modifications, exercise prescription (30-minute exercise, five times per week) and exercise video) |  |  | | | | | | |  |  |
|  |  |  |  |  | | | | | | |  |  |
|  |  |  |  |  | | | Verbal persuasion |  | | |  |  |
|  |  |  |  |  | | | | | | |  |  |
|  |  | - Booster intervention (SMS messaging) |  |  | | | | | | |  |  |
|  |  |  |  |  | | | Somatic and emotional states |  | | |  |  |
|  |  |  |  |  | | | | | | |  |  |
|  |  |  |  |  | | | | | | |  |  |

**Appendix 3**

**Figure 1**

*Flow Chart of the Study*


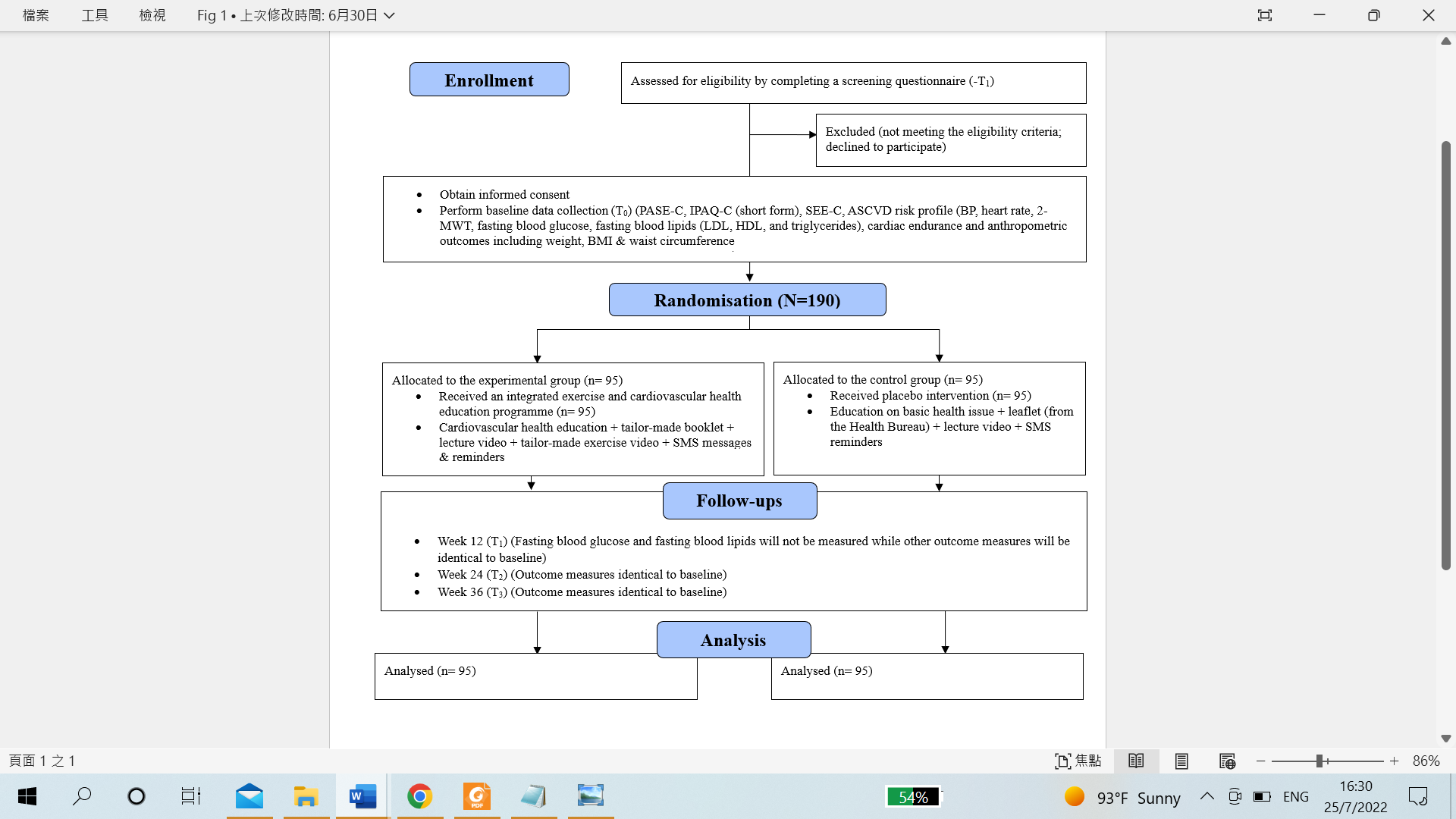


**Appendix 4**

*ASCVD Factor List (AHA, 2015; CDC, 2019; CDC, 2020; WHO, 2011; WHO, 2017)*

i) current smoker;

ii) current drinker (Women: less than one drink daily; men: less than two drinks daily);

iii) physically inactive (weekly 30-minute moderate activities of not more than five times or weekly 20-minute vigorous activities of not more than three times);

iv) diagnosed with hypertension or currently taking antihypertensive medications;

v) diagnosed with diabetes or currently taking diabetes medications;

vi) diagnosed with hyperlipidaemia or currently taking lipid-lowering medications;

vii) being overweight (BMI≧25 kg/m^2^);

viii) having a family history of coronary heart disease or stroke;

ix) currently taking anticoagulants in preventing the occurrence of ASCVD

**Appendix 5**

| Intervention components | Details  Weeks | 1 | 2 | 3 | 4 | 5 | 6 | 7 | 8 | 9 | 10 | 11 | 12 |
| --- | --- | --- | --- | --- | --- | --- | --- | --- | --- | --- | --- | --- | --- |
| Health educational session | A face-to face, group-based HE session (60-70 minutes)   - 30-minute health talk (ASCVD prevention, multicomponent exercise & peer sharing activity) - 15-minute exercise demonstration - Remaining time: exercise group practice | V |  |  |  |  |  |  |  |  |  |  |  |
| Booklet | The booklet will be provided for reference before session commencement   - PA information (advantages, exercise types, exercise intensity, demonstration pictures and a QR code for exercise video) - CV lifestyle modifications - An inquiry number for answering participant concerns | V |  |  |  |  |  |  |  |  |  |  |  |
| Multi-component exercise | 25-minute exercise: a combination of balance training, aerobic and muscle-strengthening activities using a chair and a towel   - Can be performed at home or in group setting - 5-minute warm-up and 5-minute cool-down activities - 15-minute exercises with moderate intensity - Participants are encouraged to perform the exercise twice a day (30-minute exercise dosage per day) | V |  |  |  |  |  |  |  |  |  |  |  |
| Booster intervention: SMS messaging | A total of 36 text messages sent from Week 1 to Week 12   - Three messages per week (One message on every Monday, Wednesday, and Friday) - Delivered in the daytime between 9am and 5pm - Message contents primarily cover key educational points, encouraging words and reminders of performing PA - At Week 7, a progress report regarding the interim evaluation result, accompanied with advice and positive feedback will be sent | V | V | V | V | V | V | V | V | V | V | V | V |
|  |  |  |  |  |  |  |  |  |  |  |  |  |  |

*Intervention Table*
